# Curcumin in Health and Diseases: Alzheimer’s Disease and Curcumin Analogues, Derivatives, and Hybrids

**DOI:** 10.3390/ijms21061975

**Published:** 2020-03-13

**Authors:** Eirini Chainoglou, Dimitra Hadjipavlou-Litina

**Affiliations:** Department of Pharmaceutical Chemistry, School of Pharmacy, Faculty of Health Sciences, Aristotle University of Thessaloniki, 54124 Thessaloniki, Greece; echainogg@pharm.auth.gr

**Keywords:** Alzheimer’sdisease, curcumin analogues, derivatives, hybrids, diagnosis, therapy

## Abstract

Worldwide, Alzheimer’s disease (AD) is the most common neurodegenerative multifactorial disease influencing the elderly population. Nowadays, several medications, among them curcumin, are used in the treatment of AD. Curcumin, which is the principal component of *Curcuma longa*, has shown favorable effects forsignificantly preventing or treating AD. During the last decade, the scientific community has focused their research on the optimization of therapeutic properties and on the improvement of pharmacokinetic properties of curcumin. This review summarizes bibliographical data from 2009 to 2019 on curcumin analogues, derivatives, and hybrids, as well as their therapeutic, preventic, and diagnostic applications in AD. Recent advances in the field have revealed that the phenolic hydroxyl group could contribute to the anti-amyloidogenic activity. Phenyl methoxy groups seem to contribute to the suppression of amyloid-β peptide (Aβ_42_) and to the suppression of amyloid precursor protein (APP) andhydrophobic interactions have also revealed a growing role. Furthermore, flexible moieties, at the linker, are crucial for the inhibition of Aβ aggregation. The inhibitory activity of derivatives is increased with the expansion of the aromatic rings. The promising role of curcumin-based compounds in diagnostic imaging is highlighted. The keto-enol tautomerism seems to be a novel modification for the design of amyloid-binding agents. Molecular docking results, (Q)SAR, as well as in vitro and in vivo tests highlight the structures and chemical moieties that are correlated with specific activity. As a result, the knowledge gained from the existing research should lead to the design and synthesis ofinnovative and multitargetedcurcumin analogues, derivatives, or curcumin hybrids, which would be very useful drug and tools in medicine for both diagnosis and treatment of AD.

## 1. Introduction

Globally, Alzheimer’s disease (AD) is the most common cause of dementia. According to World Alzheimer’s report 2019, over 50 million people worldwide are living with dementia, and this number is expected to increase to more than 152 million by 2050 [1]. AD is characterized by serious loss of short-term memory and impaired cognition, followed by neurodegeneration. The etiology of AD is still under research, and a lot of causes have been suggested to be correlated to the onset of AD [2]. Many risk factors have been proposed to be significant contributors to the onset of AD such as: (i) nongenetic factors such as toxins, viruses, prions, head trauma, low level of education and (ii) genetic factors such as gene mutations (AβPP, amyloid-β precursor protein; PS1, presenilin-1; PS2, presenilin-2; ApoE, apolipoprotein E; and Down syndrome) [3].

AD is mainly characterized by the accumulation of amyloid-β (Aβ) plaques (or senile plaques) and neurofibrillary tangles (NFTs) of Tau protein, in the brain [4]. Specifically, under nonphysiological conditions, oligomeric, multimeric, and fibrillar aggregates are formed progressively by the accumulation of Aβ inducing neurodegeneration [5], whereas, due to abnormal phosphorylation of the microtubules-associated tau-protein, the NFTs are gathered within the neurons [6].

Chronic brain inflammation also is a distinctive feature of AD in which the microglia, astrocytes, and, to a certain extent, neurons are thought to be strongly involved in the inflammatory process. Furthermore, the overexpression of proinflammatory mediators, such as tumor necrosis factor alpha (TNFα) and interleukin 6 (IL-6), and acute proteins are evident in different regions of an AD brain. A synergistic pattern between AD senile plaques and proinflammatory cytokines increases the neurological damage to the brain [7].

Various human diseases similar to AD, are associated with functional disorders of mitochondria. Specifically, the role of the mitochondria in the eukaryotic cells is essential. Furthermore, since they participate in a wide number of metabolic reactions and are the major source of adenosine triphosphate (ATP), they regulate numerous cellular functions, as well as proliferation, differentiation, and apoptosis. Mitochondria are simultaneously a laboratory of production and a primary target of reactive oxygen species (ROS) [8].

Furthermore, oxidative stress appears to be a major determinant of AD pathogenesis and progression [9]. High levels of oxidation products of biomacroolecules (proteins, lipids, carbohydrates, and nucleic acids) have been observed in numerous studies in AD brains and peripheral systems. It should to be mentioned that levels of antioxidant enzymes were found to be altered in brain regions of AD patients [10]. In addition, lipid peroxidation and concentrations of Fe, Cu, Al, and Hg in AD patients were found to be increased [11].

Acetylcholinesterase (AChE) plays a crusial role in AD patients. Recently, the role of neocortical acetylcholine (Ach) in spatial memory has been obtained. The discovery of the cholinergic deficit in AD underlined the role of AChE as a therapeutic target [12]. 

The fact that AD is a multifactorial disorder, with several intercorrelated pathological routes, led a huge research in the design of multitarget-directed ligand (MTDLs) [13]. MTDL, which is rationally designed to simultaneously hit multiple targets, to improve the pharmacological profiles, has been developed as a promising approach for drug discovery against AD [14]. MTDLs consist of a single or hybrid compound capable of influencing several targets or systems.

Curcumin, a natural phenolic compound extracted from the rhizome of the plant Curcuma longa, is a member of the curcuminoid family. The characteristic yellow color of the turmeric is due to the curcuminoids present in it, namely curcumin, demethoxycurcumin, bisdemethoxycurcumin, and cyclocurcumin. Curcuminoids are found in 3% to 5% of turmeric, and curcumin is the major bioactive constituent [15]. Curcumin is of great interest to researchers because it has a wide variety of bioactivities, including anti-inflammatory, antioxidant, as well as anti-AD properties [16,17]. They inhibit the formation of large toxic Aβ oligomers [18], inhibit of Aβ and tau aggregation in vivo [19], have anti-inflammatory [20,21] as well as antioxidative properties [22], and also inhibit specific enzymes such as AChE, butrylcholinesterase (BChE) [23,24], β-secretase (BACE-1) [25], and glycogen synthase kinase 3 beta (GSK3β) [26]. In addition, curcumin analogues have been found to act as metal-chelating agents [9,11,27], as well as being efficient antioxidants in mitochondria [8]. Curcumin inhibited aggregation and fibril formation of Aβ by binding to small Aβ species, [28] as well as it diminished oxidative stress [29]. It also has numerous therapeutic biochemical and molecular targets, including transcription factors, inflammatory cytokines, enzymes like β-secretase and acetylcholinesterase, kinases, growth factors, receptors, adhesion molecules, and anti-apoptosis proteins for AD [30,31]. However, due to the poor bioavailbility and selectivity of curcumin, its use is significantly limited. Curcumin’s interaction with several molecular targets, diminishes selectivity. In the last decade, researchers have focused on curcumin analogues to try to optimize the beneficial properties of curcumin against AD and improve its pharmacokinetic profile. Curcumin analogues have been designed and synthesized as multitarget anti-AD agents showing promising results in both treatment and diagnosis [32,33,34] of AD.

## 2. Curcumin Analogues and Derivatives for AD Therapy 

### 2.1. Inhibition of (Aβ)Amyloid-β Aggregation 

Sequential cleavages by BACE1 and γ-secretase (presenilin) on AβPPproduced a 40 to 43 aminoacid amyloid-β (Aβ) peptide which was found in an AD patient’s brain and it is believed that it is the main component of the senile plaques. ADAM (adisintegrin and metalloproteinase), such as ADAM10 and ADAM17 which are responsible for the constitutive and PKC (protein kinase C) regulated pathways, induces the cleavage in the middle of the Aβ sequence leading to the formation of soluble amyloid precursor protein α (sAPPα) fragment, with neurotrophic and neuroprotective properties [35]. The cleavage procedure also includes membrane proteins such as PS1 and PS2 [35]. PS1 is used as a substrate for GSK-3β. It is also included as a factor in the etiology of AD [36]. PS1 and PS2 cleave a wide number of relevant physiological substrates and their inhibition leads to a toxic situation in the nervous system, the immune system and the gastrointestinal tract [35]. Researchers have suggested the scenario to activate the α-secretase processing of AβPP as a promising alternative idea, which is not yet been estimated. 

Aβ_42_ isoform more easily aggregates and is more toxic to neurons than any other Aβ isoform, regarded as the primary toxic species in AD [37]. Thus, inhibition of Aβ accumulation is a crucial target for the treatment of AD. 

**Figure 1:**
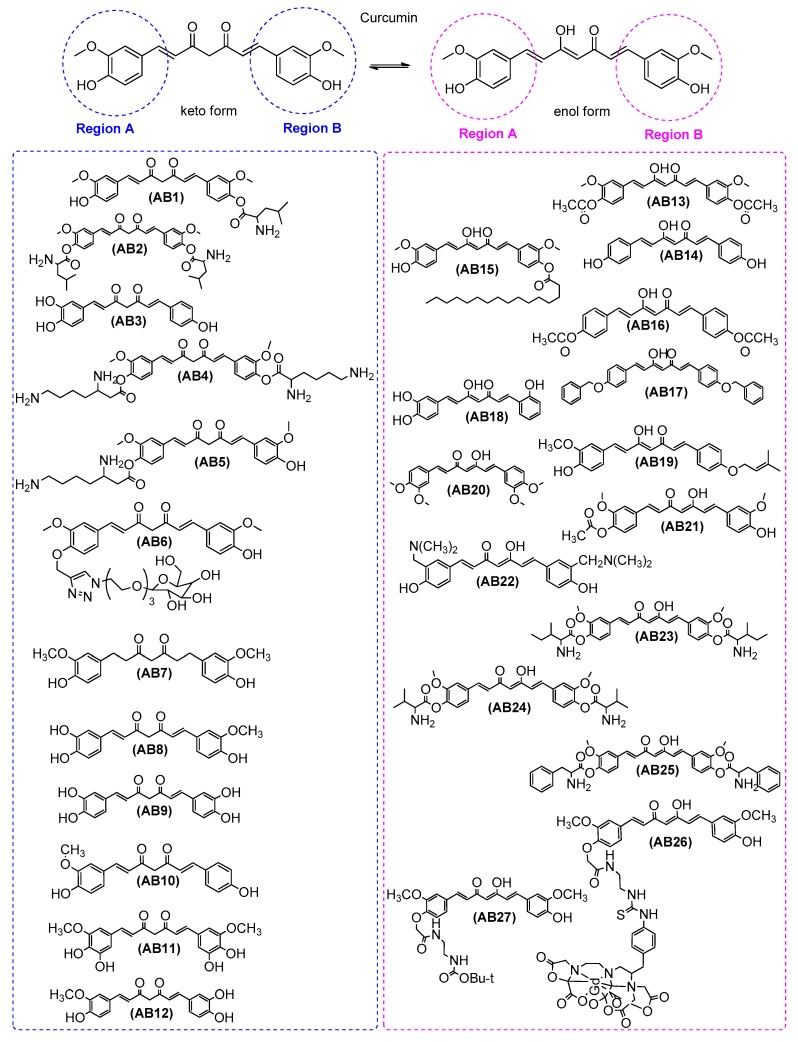
Inhibitors of amyloid-β aggregation.

Cui et al. [38] in order to mofify curcumin, used Boc-L-isoleucine to synthesize two new curcumin derivatives as inhibitors of the formation of amyloid fibrils that exhibited enhanced solubility in water. An interaction study showed that both curcumin derivatives, AB1 and AB2, could bind with hen egg-white lysozyme (HEWL) close to the tryptophan amino acid residues area. As a result, a new ground-state complexwas formed, before HEWL rendered into amyloid fibrils, inhibiting the production of amyloid fibrils. It was found that the derivative AB2significantly inhibited HEWL fibrils formation. AB2 does not conain ahydroxyl substituent which means thatthe presence of this moiety is not the only prerequisite for a curcumin derivative to be aninhibitor of the formation of amyloid fibrils (Figure 1 and Table 1). 

Wang et al. [52] found that AB4 at 20.139 mM and AB5 at 49.622 mM highly inhibited the amyloid fibrillation of HEWL. AB4 and AB5 were synthesized as two novel lysine-functionalized curcumin derivatives and their solubility in water was greatly increased as compared with curcumin. In addition, the intermolecular interaction between curcumin derivatives and lysozyme was assigned to hydrophobic interactions (Figure 1 and Table 1).

Chen et al. [54] found that poly-substituted hydroxylcurcuminoids are able to upregulate neprilysin, the most important Aβ-degrading enzyme. Thus, these compounds can be used to prevent AD. It is known that curcumin does not present this ability. The role of neprilysin is correlated with the late onset of AD since its expression level decreases with age and is inversely correlated with amyloid accumulation. Researchers have proposed four compounds, AB3, AB8, AB9, and AB11 that increase NEP activity, while curcumin does not. Treatment of APPswe/PS1dE9 double transgenic mice (B6C3-Tg(APPswe, PSEN1dE9)85Dbo/Mmjax) with AB8 or AB9 upregulated NEP levels in the brain and reduced Aβ accumulation in the hippocampus and cortex (Supplementary Data). Curcumin is well known to present poor solubility and bioavailability. However, the solubility and bioavailability of the above compounds would be greater than curcumin due to the presence of many hydroxylsubstituents on these curcuminoids (Figure 1 and Table 1).

Dolai et al. [47] succeessfully synthesized, using “click chemistry”, a water-soluble sugar derivative of curcumin with enhanced bioactivity which inhibited ∼1000 times more curcumin than the amyloid-β peptide aggregation. AB6 inhibited Aβ peptide aggregation at concentrations as low as 8 nM and lower concentrations than curcumin (Figure 1 and Table 1).

Mishra et al. [55] used rat primary hippocampal cultures and found that compound AB7, a metabolite of curcumin, showed a protective effect by reducing amyloid-β-induced toxicity. In addition, AB7 protects human neurons from oligomeric amyloid-β-induced toxicity (Figure 1 and Table 1).

Liu et al. [37] examined the effects of curcumin, AB12, and AB9 on Aβ_42_, amyloid precursor protein (APP), and BACE1 in swAPP HEK293 cells (human HEK293 cell lines overexpressing APP, Supplementary Data). They found that phenyl methoxy groups can contribute to the suppression of Aβ_42_ and to the suppression of APP. The researchers found that different curcuminoids presented different effects on the BACE1 expressions, for example, curcumin did not affect BACE1 mRNA (messenger RNA) and protein levels, AB12 suppressed BACE1 mRNA level, and AB9 suppressed both BACE1and mRNA protein levels. Among the curcuminoids tested in their study, AB9 exhibited the most potent inhibition on BACE1 expression. Thus, it seems that replacement of the methoxy group by a hydroxyl group could increase the inhibition on BACE1 expression (Figure 1 and Table 1).

Villaflores et al. [45] indicated the effects of curcumin and AB10 on amyloid-β precursor protein through the internal ribosome entry sites in order to be a potential treatment for AD. AB10, a structural analogue of curcumin, inhibited APP IRES (APP internal ribosome entry site) dependent translation initiation. It seems to be equipotent to curcumin. This result indicates that curcumin can play a role in AD pathology alleviation through the inhibition of the APP IRES-mediated translation mechanism (Figure 1 and Table 1).

Mohammadi et al. [50] studied the inhibitory activities of curcumin as a natural polyphenolic compound and diacetylcurcumin AB13 as a synthetic derivative of curcumin on the amyloid fibrillation of HEWL. Both amyloid fibrillation and binding results indicated that interaction of AB13 with HEWL was stronger than that of curcumin, and amyloid fibrillation of HEWL was inhibited more effectively by AB13 than curcumin. These results support the concept that both acetyl groups of AB13, as well as the hydroxyl groups of curcumin are similarly interacting with amino acid residues of protein and interrupting amyloid structure. Thus, both curcumin and its synthetic derivative AB13seems to prevent the lipophilic aggregation of protein and its toxic intermediates via different groups of curcuminoids scaffold including: the β-diketone moiety, the phenolic OH groups, the acetyl groups, the benzene ring, the hepta-diene group, and the substituents on the benzene ring (Figure 1 and Table 1).

AB14 which is a natural poly-hydroxyl-subsututed phenolicderivative and AB16 which is a synthetic derivative of curcumin were studied by Mohammadi et al. [51] for their activities on the amyloid fibrillation of HEWL. It seems that AB14 interacts with HEWL stronger than AB16 whereas AB14inhibits amyloid fibrillation of HEWL higher than AB16in correlation tothe higher binding activity of AB14 with HEWL. The inhibitory activity of AB14, a phenolic polycyclic structure of low molecular weight is related to the contribution of different types of physicochemical properties. The implicated properties, which stabilize the binding to the intermediate structures of lysozyme and finally suppress the amyloid formation, arethehydrogen bonding, the lipophilic interactions, and the aromatic stacking. AB16 presents considerable interaction with lysozyme. The resultssupport the existence of a significant relationship between the anti-amyloidogenic activity of both curcuminoids, as well as the described interactions with lysozyme. The hydrogen bonding between the AB14 and AB16 with tryptophan 63 (Trp-63) stabilizes the intermediate structures of lysozyme and diminishing the amyloid formation (Figure 1 and Table 1).

Qi et al. [60] synthesized a novel derivative, a palmitic acid curcumin ester, AB15. This curcumin derivative, cultivated on the membranes of neurocytes, seems to be able to inhibit the direct interaction between Aβ and the cellular membrane. AB15 was compared to curcumin, after morphological analyses of the cell shape. The protection results induced by AB15 were better. Curcumin and AB15 were examined for their binding affinities on Aβ. This experiment provided information with respect to their ability to inhibit the direct interaction between Aβ and cell membranes. AB15decreased the direct interaction between Aβ and cell membranes and induced greater neuroprotection against Aβ than curcumin (Figure 1 and Table 1).

Di Martino et al. [26] studied the inhibitory activity of AB17 on BACE-1 (IC_50_ = 40 nM). Docking simulations verified that curcumin’s structural motif was appropriate for BACE-1 inhibition, The structural motifs, which strongly inhibited BACE-1 combinedas A ring, the 4-hydroxy-3-methoxyphenyl moiety of curcumin, and as B ring, the 4-benzyloxyphenyl or para-tolyl with IC_50_ values of 0.97 and 0.14 μM, respectively. A remarkable decrease in potency (two orders of magnitude) was observed by the corresponding diketo tautomer. Docked pose of AB17 at BACE-1 binding site was in good agreement with the reported activity. The results from the docking analysis, delineate that the best binding affinities are related to the benzyl rings of AB17-based analogues, since these could solely be decorated with small substituents, e.g.,a fluorine atom (Figure 1 and Table 1).

Hitoshi Endo et al. [53] found that AB18 is the ideal in vitro amyloid-β aggregation inhibitor among synthesized curcumin analogues. AB18 also has water solubility that is 160 times higher than curcumin. The researchers applied SAR studies of curcumin analogues and found that a catechol motif pharmacophore wascapable of inhibiting Aβ aggregation. Furthermore, they described the following two new approaches for improved water solubility of curcumin analogues: (a) reduction of molecular planarity and (b) use a β-diketone moiety. The presence of hydroxyl group as a substituent on the aromatic ring of curcumin analogues seems to be significant in connection to a ketone group stabilizing the torsion of the o-phenol ring and neighboring olefin (Figure 1 and Table 1). 

The docking results by Konno et al. [25] indicated that AB18was acting as a new nonpeptidyl inhibitor of BACE-1. Two polar phenolic hydroxy groups and a ketone of AB18 were involved in the hydrogen bonding interactions (glycine Gly230 and glutamate Glu339). AB18 did not directly interact with the active site of BACE1 but with a cylinder-shaped space near the P3 pocket, which could be a new potential target for the design of new anti-BACE1 agents. It seems that it is a noncompetitive inhibitor and its mode of action is partially correlated with the substrate’s binding to the P3 pocket. In addition, SAR results showed that aromatic hydroxy groups and an alkenyl linker are significant structural characteristics for BACE-1 inhibitors. Both enzymatic kinetics assays, as well as docking studies, anticipated a noncompetitive inhibition mode of action. This study indicated that free hydroxy groups in place of bis-phenols were preferable for the activity but were not potently effective as compared with those of curcumin. Ketones and double bonds simultaneously seem to be in the spacer essential motifs. The corresponding reduced compounds showed no inhibitory activities due to the high degree of flexibility. Thus, a planar sp^2^ carbon unit seems to be a significant inhibitor. The rigid structure of the spacer and the phenolic hydroxy moieties cooperate on BACE1 inhibition, supporting a specific conformation. In particular, the o-phenol motif keeps both planar scaffold for inhibition and water solubility. The replacement of phenol rings with indole and pyrrole groups also leads to effective alternative agents for inhibition and water solubility and is expected to occur in the interaction with hydrogen bonds for BACE-1 (Figure 1 and Table 1).

Bisceglia et al. [18] synthesized prenylated curcumin analogues as multipotent tools to tackle AD. AB19 was found to inhibit the production of large toxic Aβ oligomers towards smaller nontoxic and insoluble fibrils. AB19 presented potential abilityto bind Aβ structures of various complexity. Molecular dynamics and molecular docking studies supported the biological results. In particular, AB19 turned out to be less toxic than curcumin. AB19, endowed with the hepta-trien-3-one linker, was the most effective agent in slowing down the formation of toxic Aβ oligomers. The anti-amyloid effects of AB19 as compared with those of curcumin were evidently corroborated by molecular docking and steered molecular dynamics. These studies revealed the significance of the lipophilicityin simulation of Aβ structures of increasing complexity. The experimental biological results taken togetherwith the molecular dynamics and molecular docking studies defined the major role of the curcumin motif to secure anti-amylodogenic activity, especially when aryl substituents are present such asthe 4-hydroxy, 3-methoxy, and prenyloxy groups (Figure 1 and Table 1).

Orlando et al. [46] synthesized curcumin analogues as improved inhibitors of amyloid beta oligomerization. The simple substitution of the para-hydroxy group on curcumin with a methoxy substitution (AB20) improved inhibitory function by six- to seven-fold over that measured for curcumin, making AB20 a potent lead analogue for anti-Aβ aggregation activity. The prerequisites for anti-Aβ aggregation activity are given as follows: (i) One enone group at least in the linker between aryl rings; (ii) an unsaturated carbon linker between aryl rings, saturated carbon linkers did not improve the activity; and (iii) methoxy and hydroxy substituents in the meta- and para-positions on the aryl rings. The optimalstructural characteristics for the design of lead inhibitors include either (a) meta- and para-substituted methoxy and hydroxy groups reversed from that of curcumin, or (b) methoxy or hydroxysubstituents in both positions. The combination of thepara-hydroxy group on curcumin with a methoxy substituent improved inhibitory function by six- to seven-fold over curcumin (Figure 1 and Table 1).

In a study by Lakey-Beitia et al. [7], curcumin derivatives were synthesized by etherification and esterification of the aromatic region. Their findings suggest that the novel curcumin derivative AB21 is an active therapeutic compound for the treatment of AD. AB21 exhibited in vitro a strong anti-aggregation effect which was higher than that of curcumin. It should be notedthat the volume of the substituents in the scaffold of curcumin derivatives had a negative effect and decreased bioactivity. Furthermore, structure–activity relationship studies (SAR) have indicated that curcumin derivatives etherified with small groups at both sides of the molecule lose the anti-aggregation activity. Etherification at only one side of curcumin maintained the bioactivity. Acetylation at only one side of the molecule led to an analogue with higher biological activity (AB21). Monofuctionalized diester analogues showed a decreased or null bioactivity as compared with curcumin. This decrease was associated with the complexity and length of the analogue. The presence of bulky groups in monofuctionalized diester derivatives negatively influenced the activity. Bifunctional diester exhibited a reduced biological activity. It has been suggested that phenolic compounds, such as curcumin, are able to produce anti-aggregation activity due to π−π interactions and the formation of hydrogen bonds between the peptide and the phenolic rings (Figure 1 and Table 1).

Fang et al. [16] synthesized dimethylaminomethyl-substituted curcumin derivatives and found that AB22 could effectively inhibit the Aβ self-aggregation in vitro. AB22 showed a good stability while curcumin did not. 

Curcumin can be rapidly metabolized in vivo. The main metabolites are curcumin glucuronides and sulfates through the phenolic hydroxy groups. The presence of dimethylaminomethyl groups, due to steric hindrance, can prevent the metabolism and prolong the half period time of the target compounds (Figure 1 and Table 1).

Narasingapa et al. [35] presented several new α-secretase activators derived from modified curcumin-based compounds. They found that conjugates of curcumin with aminoacids isoleucine, valine, and phenylalanine, i.e., AB23 (curcumin-isoleucine), AB24 (curcumin-valine) and AB25 (curcumin-phenylalanine) inducedthe constitutive activity of α-secretase and increased ADAM10 immunoreactivity. In vitro assayswere performed under conditions mimicking the PKC/muscarinic receptor-regulated pathway. The results displayed different modes of activation. All the data were analyzed and indicated new lead compounds as potent and stable activators of α-secretase. The results showed the presence of a discrimination ability. Thus, some molecules are capable ofpromotingthe constitutive and others the regulated α-secretase pathways (Figure 1 and Table 1).

Ouberai et al. [61] designed a new series of amyloid inhibitors to interfere with Aβ aggregation. The compounds were synthesized and evauated in vitro. For the curcumin synthesis or the KLVFFA peptide, a copper(I)-mediated azide–alkyne catalytic cycloaddition was tied on a constrained cyclopeptide moiety. The derived conjugates were very potent inhibitors of the amyloid fibril formation. This research underlines the significant influence on the inhibition of amyloid fibril formation by making a new scaffold using a bulky group in combination with Aβ-recognition domains (Table 1).

Kochi et al. [29] studied the inhibition of metal-free and metal-induced amyloid-β aggregation induced by curcumin derivatives. From these samples, TEM images revealed shorter, thinner, and less dense fibrils upon curcumin, AB27 (Cur-L), or AB26 (Gd-Cur) treatments. The promising increase in solubility of AB26 in water and its behavior withmetal-free Aβ and metal−Aβ in vitroshow that the presented modifications of curcumin are worthy for improvement of its properties. The phenolic groups of curcumin could be essential for Aβ interaction, as well as for radical scavenging. AB26seems to be a potent lead for the inhibition of metaltriggered Aβ aggregation (Figure 1 and Table 1).

**Figure 2:**
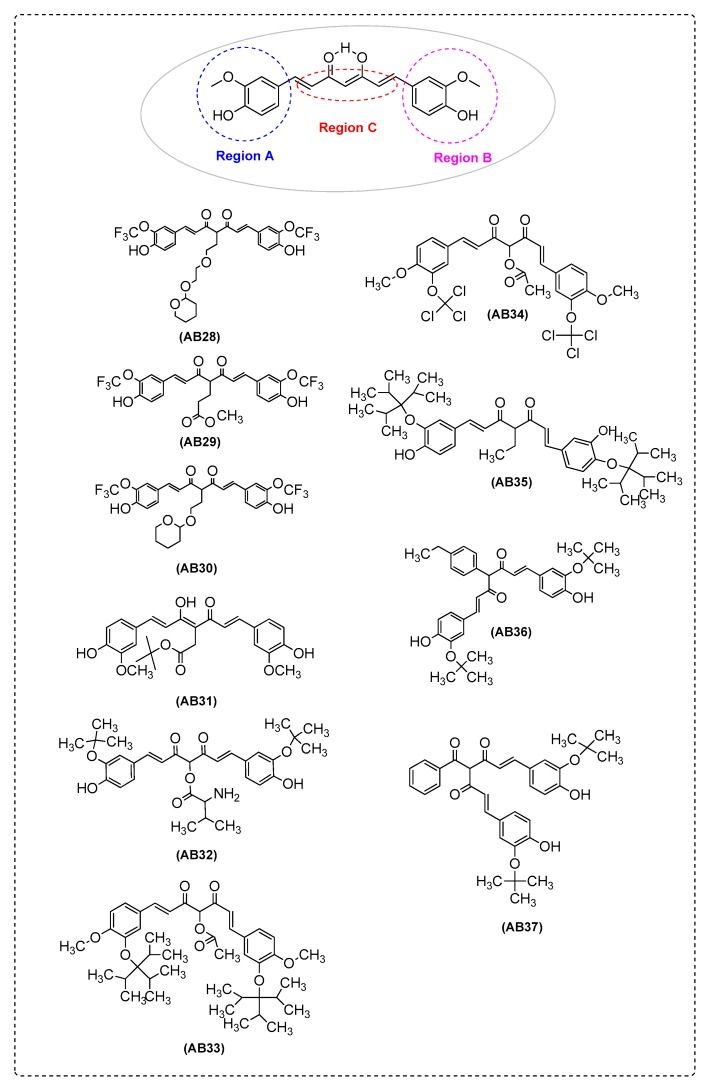
Inhibitors of amyloid-β aggregation.

**Figure 3:**
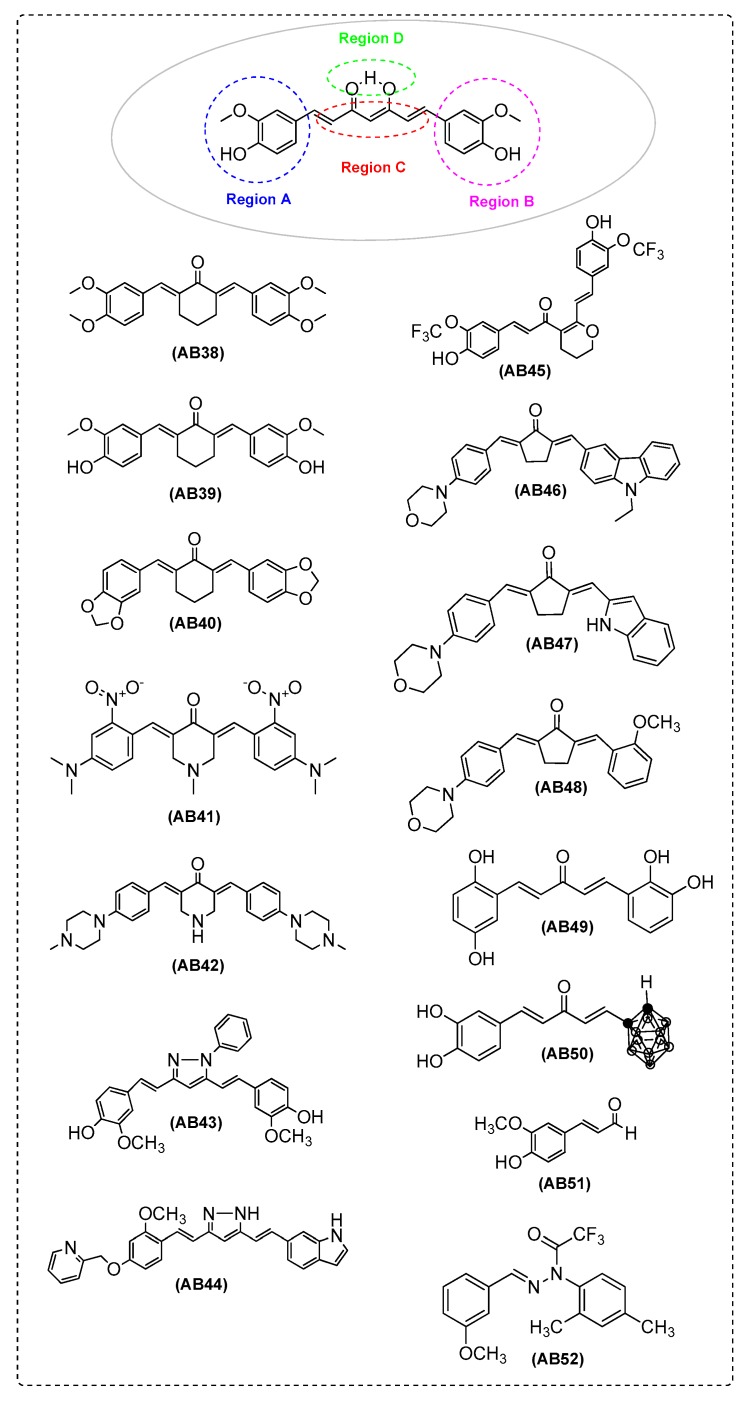
Inhibitors of amyloid-β aggregation.

Yanagisawa et al. [30] synthesized four new curcumin derivatives which highly inhibited Aβ aggregation in SH-SY5Y cells (human neuroblastoma cell line, Supplementary Data). They proposed AB28, AB29, AB30 (Figure 2), and AB45 (Figure 3) as therapeutic candidates for preventing AD. The in vitro results showed the curcumin derivative with a 1,7-bis(4′- hydroxy-3′-trifluoromethoxyphenyl) groupwas more potent than the derivative with a 1,7-bis(4′-hydroxy-3′-methoxy phenyl) group, suggesting that trifluoro methoxy groups on aromatic rings are favorable groups for inhibiting Aβ aggregation. In addition, curcumin derivatives that showed effects on Aβ aggregation have at least one hydroxy group as a substituent on the aryl ring groups in their structure. The absence of this group from the curcumin is correlated with the disappearance of any effects on Aβ aggregation. The results supported that the presence of hydroxyl groups on the aromatic groups in curcumin derivatives are essential to inhibit Aβ aggregation. The curcumin derivatives AB28, AB29, AB30, and AB45 are substituted at the C-4 position. Taken together, they assumed that the combination of 1,7-bis(4′-hydroxy-3′-trifluoromethoxyphenyl) groups and a suitable substituent at the C-4 position are crucial for the therapeutic effect of curcumin derivatives (Table 1). 

Furthermore, Yanagisawa et al. [44] tested the effects of AB29 on AD pathology in APPswe/PS1dE9 double transgenic mice (Supplementary Data). AB29 significantly attenuated the cell toxicity of Aβ. These results support that AB29 has potential for preventing AD (Figure 2 and Table 1).

Ferrari et al. [40] proposed that AB31 is the best candidate for the development of new curcumin-based analogues to treat AD, due to its multifaced intrinsic characteristics which include: (i) it is able to inhibit Aβ_1–40_ aggregation; (ii) it seems that it is tightly bound to Cu(II) giving more stable complexes with respect to curcumin, reducing metal concentration in the free form and, consequently, limiting Aβ_1–40_ aggregation; and also (iii) AB31 is more stable than curcumin in physiological conditions, suggesting higher bioavailability. AB31 was derived by the introduction of a t-butyl ester group attached to a methylenic linker on the the β-diketo moiety of curcumin frame (Figure 2 and Table 1). 

Aswathy et al. [57] designed novel amyloid-β aggregation inhibitors using quantitative structure–activity relationship (QSAR); pharmacophore modeling; molecular docking; and absorption, distribution, metabolism, excretion and toxicity (ADMET) prediction models. They selected six lead molecules which indicated the best results, AB32, AB33, AB34, AB35, AB36, and AB37 (Figure 2).

Specifically, the two-dimensional (2D) QSAR model gave the following statistical values: the square of correlation coefficient (R^2^) = 0.9086 and standard errors of estimate (SEE) = 0.1837. The outcome from the cross-validation procedure was cross-validated R^2^ (Q2), which was used as a criterion of both robustness and predictive ability of the model. For external validation, the parameter estimated predictive correlation coefficient (R^2^_pred_) was used, to verify the predictive ability of the model on the test set. In addition, the parameter non cross-validated correlation coefficient (R^2^_ncv_) was used.

pIC_50_ = 5.17173(± 0.03835) − 5.687(± 0.60642)MDEC – 44 + 1.29883(± 0.2094)WK.eneg − 0.20612(± 0.1117)ExtFP728− 0.36098(± 0.09881)GraphFP295 + 1.20961(± 0.12137)GraphFP912+ 0.0091(± 0.0057)PEOE_VSA + 4

MDEC-44 is the molecular distance edge between all quaternary carbons. Similarly, ExtFP728 and GraphFP295 also shows a negative contribution. WK. eneg is the non-directional weighted holistic invariant molecular (WHIM), weighted by Mulliken atomic electronegativities and the descriptor, GraphFP912 positivelyinfluences the biological activity. The hologram quantitative structure–activity relationship (HQSAR) study (Q^2^ = 0.615, R^2^_ncv_ = 0.931, and R^2^_pred_ = 0.956) illustratesthe important molecular fingerprints for inhibition. Contour maps of three-dimensional (3D) QSAR models, comparative molecular field analysis (CoMFA) (Q^2^ = 0.687, R^2^_ncv_ = 0.787, and R^2^_pred_ = 0.731), and comparative molecular similarity indices analysis (CoMSIA) (Q^2^ = 0.743, R^2^_ncv_ = 0.972, and R^2^_pred_ = 0.713) underline the robust character of the taken models and highlight the significant physicochemical properties (steric, electrostatic, and hydrogen bond acceptor) for interaction with the receptor site.

The undertaken molecular docking studies of the curcumin derivatives describe the prerequesite structural characteristics for the exploration of the pharmacophore features of Aβ aggregation inhibition. They have presentedthe significant interactions that take place between the amino acids at the site of action. In addition, novel chemical structures have been analyzed with the aid of inverse QSAR studies and information has been obtained for the Aβ aggregation inhibitory activities.

Pharmacophore analysis showed that pharmacophore is a combination of steric, electrostatic, H-bond donor, H-bond acceptor, and hydrophobic structural characteristics which are necessary to ensure the optimal molecular interactions with the biological target. The results show that the generated pharmacophore model requires two hydrophobic and aromatic features, two hydrogen bond donor functions, and one hydrogen acceptor function. 

The prediction of the site of metabolism (SOM) and the ADME properties provide information useful for the drug design in order to improve the metabolic properties of the molecule. The best SOM among the compounds AB33, AB34, AB35, AB36, and AB37 is the themethylene group (–CH_2_–) positioned between the two carbonyl groups where, during metabolism, an aliphatichydroxylation occurs. The best SOM for AB32 is the methylene group attached to the –NH_2_ group. For compound AB37, as second SOM is ranked the aromatic hydroxylation on the double bonds of the aromatic ring in vicinity to the oxygen atom. In compounds AB33 and AB34, the methoxy group is ranked as the second SOM.For the methoxy moiety, O-dealkylation reaction is also regarded for metabolism (Table 1).

Liu et al. [62] designed, synthesized, and biologically characterized a series of bivalent ligands containing curcumin and cholesterylamine, which inhibited the formation of amyloid-β oligomers (AβOs) in MC65 cells (a human neuroblastoma cell line, Supplementary Data). The bivalent ligand, with its spacer (length of 17 atoms), is the most potent (EC_50_ of 0.083 ± 0.017 μM) (Table 1).

Ramshini et al. [48] found that AB38, AB39, and AB40 inhibited HEWL aggregation and inhibited the cytotoxicity of aggregated HEWL in MCF7 cells (a breast cancer cell line, Supplementary Data). The findings were found to be dosedependent. Docking results demonstrated that the compounds are bound to lysozyme and occupy the whole active site groove. The physicochemical properties and structural characteristics ofAB38, AB39, and AB40 present similarities to curcumin. Phenyl ring replaceddiketone moiety of curcumin, whereas various substituted groups were placed on the aromatic moiety. This study showed that compounds lacking this group were still more or less active. The tested compounds were more rigid structures. A sp^3^-hybridized carbon was missingin their backbone. Moreover, the methoxy substitution of hydroxy groups on AB38 and the absence of hydroxy groups on AB40 improved their bioactivity. Especially, compound AB40 was more effective (in comparison to AB38 and AB39) (Figure 3 and Table 1).

In Bukhari et al.’s [58] study, AB41 was found to be the most protective agent (87%, at a concentration of 100 mM) against Aβ-induced neuronal cell death in PC12 cells (rat pheochromocytoma cell line, Supplementary Data). Among the tested compounds, the analogues possessing 2-nitro and 4-dimethylamine groups exhibited extremely high protective activities, while a diethoxymethyl group at position 4 of the rings led to asignificant loss. Aβ-induced cytotoxicity was decreased by the insertion of a pyrolidine ring at position 4. The presence of 4-piperidone or *N*-methyl-4-piperidone as spacerled to more protection as compared with all the other compounds. These results suggested that the novel α, β-unsaturated carbonyl-based compound, AB41, can reduce cell damage caused by Aβ-induced cytotoxicity (Figure 3 and Table 1).

Chen et al. [11] verified that AB42 can decrease or inhibit the β-sheet aggregation and the fibril formation (IC_50_ = 2.5 μM and 90% inhibition at a concentration of 50 μM). A SAR analysis for all the synthesized analogues were performed, suggested that the introduction of flexible moieties at the linker is crucial for the inhibitory potencies of the compounds against Aβ aggregation (Figure 3 and Table 1).

Kotani et al. [59] found that in SH-SY5Y cells (Supplementary Data) AB43 did not inhibit β- or γ-secretase activity. AB43 stimulatedexpression of the ER chaperone glucose-regulated protein 78 (GRP78) and increased the formation of the AβPP/GRP78 complex. These findings support the fact that AB43lowers intracellular AβPP traffickingand suppresses the production of Aβ. Therefore, the curcumin derivative AB43 reduces both Aβ_40_ and Aβ_42_ production (Figure 3 and Table 1).

Okuda et al. [41] synthesized curcumin derivatives and evaluated their inhibitory activities against Aβ aggregation. The more potent aggregation inhibitor was AB44 with IC_50_ values of 1.2 μM for Aβ aggregation. In addition, AB44 presented a better pharmacokinetic profile and pharmacological efficacy in vivo ascompared withcurcumin, making it suitable as a drug for AD (Figure 3 and Table 1). One year later, Okuda et al. [19] investigated the inhibitory activity of AB44 on Aβ aggregation in SH-SY5Y cells (Supplementary Data) and the cytotoxicity. AB44 improved cognitive dysfunction and decreased Aβ aggregation in brains of senescence-accelerated mouse prone 8 (SAMP8) (Figure 3 and Table 1).

Zhai et al. [9] synthesized new asymmetric curcumin analogues which were evaluated as potential multifunctional agents for the treatment of AD. Compounds AB46, AB47, and AB48 showed high activity with 92.9%, 90.8%, and 91.8% inhibition ratio of self-mediated Aβ_1-42_ aggregation, respectively. In addition, compounds AB46, and AB48 exhibited high disaggregation ratios of Aβ_1–42_ aggregation fibrils, i.e., 84.5% and 83.2% respectively. AB48 could reduce or retard β-sheet structural formation and only slightly influence the content of β-helix structure of the peptide. The results from an SAR analysis showed that therigidity of the bridge between the two aromatic moieties is more preferable, as well as the expansion of the aromatic rings. The presence of an electron-releasing group on the aromatic ring was found to be more favorable for the inhibition of Aβ_1–42_ aggregation as compared with an electron withdrawing group. Methoxy substitution on aromatic rings led to derivatives with higher inhibition, whereas compounds with nitro substitution presented decreased activity. For compounds with the same substitution on the aromatic ring, the position of the substitution affected the result. For compounds with electron-releasing groups, the potency range was as follows: *ortho >meta >para.* On the contrary, compounds with electron-withdrawing groups followed the reverse order, *para > middle >ortho*. These results indicate that substituents with large conjugated structure or electron-releasing groups, or high cloud densityimprove the activity (AB46, 91.8% and AB47, 90.8%) (Figure 3 and Table 1).

Hotsumi et al. [39], in their research on curcumin analogues, analyzed their results of amyloid-β aggregation inhibition and carried out a SAR study. They observed that the C5-monoketone type curcumin analogue, AB49, presented satisfactory water solubility, low cytotoxicity andsignificantlyinhibited theanti-amyloid-β aggregation. This scaffold was taken by optimization of the o-phenol and the olefin linker. Docking results showed that AB49 interacted with the amino acid moieties of Aβ fibrils (Figure 3 and Table 1).

Azzi et al. [49] replaced the β-diketone moiety of curcumin by a carbonyl group and substituted one of the two aromatic rings with an ortho-carborane. Thus, they synthesized and evaluated a new class of boronated monocarbonyl analogues of curcumin (BMAC) for amyloid-β disaggregation activity. AB50 presented two hydroxyl moieties and showed better efficiency. The presence of a second –OH group enhanced the binding. The findings from the HEWL fibril aggregation support the concept that the presence of at least one aromatic group is essential for the inhibitory efficiency of these derivatives. Finally, the presence of boron atoms in the carborane cage support the boron neutron capture therapy (BNCT) as a radiative boost to enhance fibril disaggregation (Figure 3 and Table 1). 

Hui-Chien Lee et al. [56] studied the protective effect of curcumin analogues in Aβ-induced paralysis in GMC101 caenorhabditis elegans. At 100 μM, analogue AB51 and curcuminoffered protection from Aβ toxicity. Skn-1 (the nematode ortholog of Nrf2) mRNA was significantly elevated in nematodes treated with curcumin and AB51, indicating SKN-1/Nrf (nuclear factor erythroid 2-related factor) activation as a possible mode of action. The above findings suggest analogue AB51 asa new lead against Aβ toxicity (Figure 3 and Table 1).

In Chen et al.’s [42] publication, a limited SAR analysis of AB43 showed that the hydroxyl groups are not necessary for activity. On the contrary, activity was maintained by inserting two methyl groups in the nitrogen-associated aromatic ring of AB43. To improve the pharmacological properties and delineate the role of the two aromatic rings connected by a nitrogen containing bridge for activity, a large number of molecules were synthesized. The best Aβ anti-aggregation activity was presented by AB52 [43] derived from AB43. AB52 is broadly neuroprotective and enhances long-term potentiation and memory. In addition, AB52 prevents memory deficits in an AD animal model and reduces soluble Aβ levels in the hippocampus (Figure 3 and Table 1).

**Figure 4:**
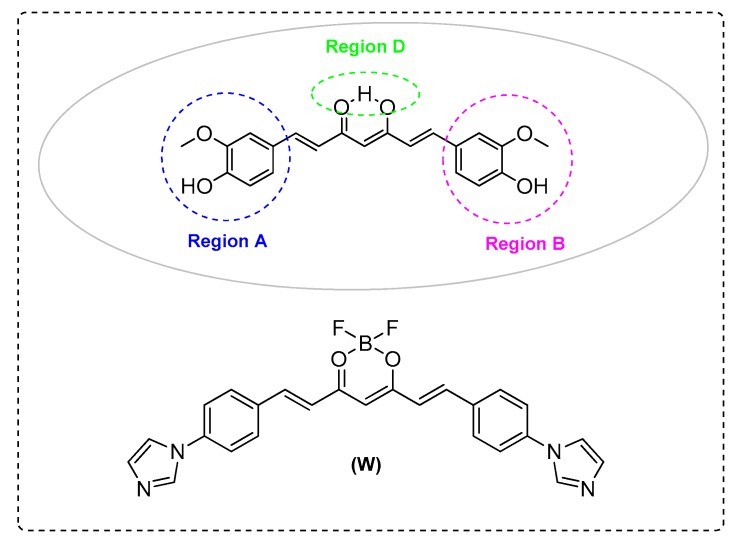
Inhibitors of amyloid-β aggregation.

Wan et al. [64] showed that a curcumin analogue (W) significantly decreasedAβ aggregation in lowers oligomeric amyloids in the cells and diminishedAβPP’s maturation in the secretory pathway. The same behavior was followed by curcumin. The analogue upregulated β-secretase and inhibited γ-secretase by decreasing BACE1 protein levels. All these data reveal mechanisms of a promising curcumin analogue, which strongly supports its development as a potential therapeutic for AD (Figure 4 and Table 1).

#### 2.1.1. Mixed Curcuminoids and Amyloid-β Aggregation

**Figure 5:**
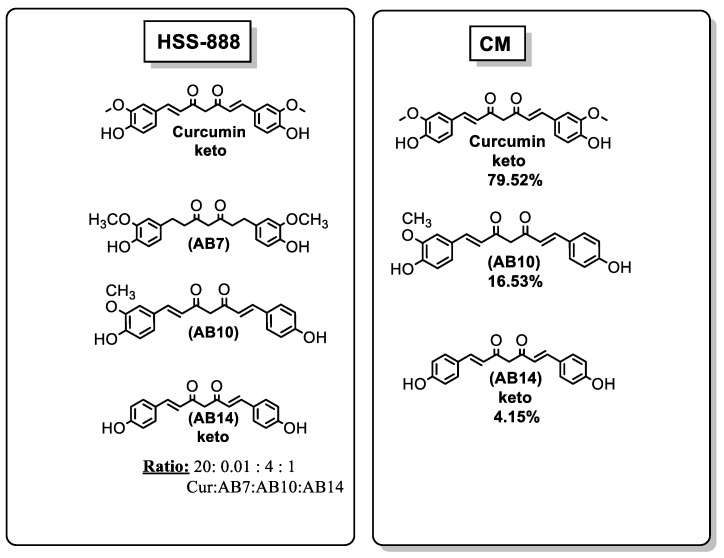
Mixed curcuminoids having anti-amyloid properties.

Shytle et al. [70] found that a curcumin extract (HSS-888) inhibited Aβ_1–42_ aggregation in vitro and Aβ_1–42_, Aβ_1–40_ generation from SweAPP N2a cells (Swedish mutant APP overexpressed N2a, Supplementary Data).HSS-888 was enriched in curcuminoids, curcumin, AB10, AB14, and AB7 in an approximate ratio of 20:4:1:0.01. The extract contained 72% curcuminoids and 28% turmerones (direct analysis in real-time mass spectrometry DART TOFMS) (Figure 5).

Ahmed et al. [71] studied the effects of curcuminoid mixture (16.53% AB10, 4.15% AB14, and 79.52% curcumin) and of each constituent on spatial learning and memory in a rat modelof amyloidbeta (Aβ) infusedpeptide. The CM mixture induced an increase in rats’ memory (Figure 5).

#### 2.1.2. Interaction of Curcumin Analogues and Derivatives with Aβ-Fibrils

Yanagisawa et al. [28] studied the relationship between the tautomeric structures of curcumin derivatives and their Aβ-binding activities. It seems that keto-enol tautomers were selectively highly bound to Aβ aggregates, whereas no binding was noticed to Aβ monomers. Thus, the results support that during the binding to Aβ aggregates, the curcumin derivatives appear mainly as enols. The enolization is a novel modification for the design of amyloid-binding agents.

Randino et al. [72] studied the structural interactions of amyloid-β peptide with single curcuminoids. Their computer-aided studiessuggested a model for the Aβ interaction of curcuminoids. The constrained “semi-folded” conformation was crucial for the interaction with Aβ. The provided pattern is analogous to thatcorrespondinglyobserved in α-helical coiled-coil peptide structures. This approach better explainsthe anti-Alzheimerin vitro and in vivo activity of curcuminoids, suggesting optimized drug-like analogues as a new choice for the rational design.

Useful information was reported by Masuda et al. [73] from nuclear magnetic resonance spectroscopy (NMR) two-dimensional NMR (2D NMR) and 2D DARR methods. The undertaken ^13^C–^13^C cross peaks suggest that curcumin interacts with the 12th and 17th to 21st amino acids included in the β-sheet structure in the Aβ_42_ fibrils. It should to be noted that aromatic carbons in the vicinity of the methoxy or hydroxy groups of curcumin presented definite cross peaks with the Aβ_42_ fibrils. These findings support that these moieties of curcumin are implicated in its interaction with the Aβ_42_ fibrils. 

Airoldi et al. [5] studied curcumin derivatives as new ligands for Aβ peptides and found that these compounds present favorable physico-chemical properties. We must consider also that the pyrazole ring as an appendage gives opportunities to be linked to other moieties, for possible future applications of these derivatives as theranostic agents of Aβ-related disease. 

An attempt to increase affinity to the Aβ fibrils was made by Mourtas et al. [74] who producednanoliposomes conjugated with curcumin derivatives. The main idea was to maintainthe stability of the planarity, which is aprerequisite for the interaction with Aβ. In order to succeed chemically, Mourtas et al. [74] used the “click chemistry” methodology. This decoration maintained thestablility ofthe required structural characteristics. These nanosized curcumin liposomes showed sufficient integrity and stability, as well as significant high binding affinity for Aβ_1–42_ fibrils (1–5 nM). Thus, they could be potentially very useful for diagnostic or therapeutic purposes.

### 2.2. Inhibition of Tau Aggregation 

NFTs have also been implicated in AD. Molecular analysis has revealed that these abnormal inclusions in cell bodies and proximal dendrites contain aggregates of hyper-phosphorylated isoforms of tau, a microtubule-binding protein that is normally soluble. Tau plays a key role in intracellular transport, particularly in axons, by binding to and stabilizing microtubules. In a diseased neuron the tau protein becomes hyperphosphorylated [3].

It has been shown that inhibitors of tau aggregation were prepared by the replacement of the β-hydroxyenone moiety of curcumin with substituted pyrazoles. 4-Nitrophenyl- and 3-nitrophenyl-substituted curcumin pyrazoles displayed inhibition of tau aggregation inhibition at low micromolar concentrations. Electron withdrawing substituents on the N-aryl pyrazoles increased 100-fold the inhibition of tau aggregation as comparedwith the N-phenylpyrazole. Similar SAR results weretaken for the disaggregation of tau protein. The nitro-substituted derivative was 18- to 70-fold more active than N-phenylpyrazole against the tau depolymerization [75].

Okuda et al. [76] found that AB44 decreased the aggregated tau and blocked the onset and induction of neural abnormalities in vivo, acting as a novel inhibitor of tau aggregation. AB44 showed inhibitory effect on tau aggregation in the spinal cord. In addition, AB44 also improved the motor dysfunction (Figure 3). The same research group [41], in 2017, investigated the therapeutic effects of AB44 on cognitive dysfunction via dual inhibition of Aβ and tau aggregation in vivo. AB44 ameliorated cognitive dysfunction and reduced the amount of aggregated tau and Aβ in brains of SAMP8. AB44, as a candidate drug for AD, presents a good pharmacokinetic profile [19]. 

Villaflores et al. [45] studied the effects of curcumin and AB10 on amyloid-β precursor and tau proteins through the internal ribosome entry sites. AB10 was observed to inhibit the phosphorylation of both tau pS262 and pS396 (Figure 1).

Yanagisawa et al. [77] found that AB29 inhibited cognitive impairment and tau accumulation in a mouse model of tauopathy, rTg4510 (express a repressible form of human tau, Supplementary Data) (Figure 2).

Furthermore, Dolai et al. [47] synthesized sugar-curcumin conjugate, AB6, which inhibited tau peptide aggregation at concentrations as low as 0.1 nM.

It is known that the blockage of the inhibitory phosphorylation of GSK-3β kinase increases tau phosphorylation [78]. Thus, Di Martino et al. [26] tried to synthesize a GSK-3β inhibitor. All curcumin-based analogues showed micromolar activities. Among them, the para-methoxy analoguewas found to be the most active (IC_50_ = 0.53 μM). Replacement of a side aryl ring of the curcumin scaffold by a para-benzyloxyphenyl or a para-tolyl group led to better inhibition of GSK-3β. Moreover, AB17, bearing a para-benzyloxyphenyl moiety, showed inhibitory activity (approximately 2 μM). The AB17-based fluorinated subset (IC_50_ values ranging from 8.30 to 16.99 μM) was found to be slightly more active, or as active as curcumin (Figure 1).

### 2.3. Anti-Neuroinflammatory Activity

In the central nervous system (CNS) microglia constitute the principal component of immune cells which are activated responding to brain injuries, damages, or diseases. As a consequence, they release neurotoxic factors, i.e., proinflammatory cytokines, for exmaple, TNF-α, interleukin 1β (IL-1β), IL-6, as well as nitric oxide (NO). Among these mediators, NO is the product of the inducible NO synthase (iNOS). NO induces neurotoxicity. It is known thatthe NO production by activated microglia is implicated in several brain dysfunctions such as multiple sclerosis, cerebral ischemia, and AD, whereas inhibition of NO production provides significant neuroprotection [79].

Tocharus et al. [79] suggested that AB3 and AB9 are the two most potent compounds that inhibited NO production. The analogues AB3 and AB9 were two-fold more active than the parent curcuminoids curcumin and AB10, respectively.AB3 and AB9could be used as potent alternative therapeutic compounds for neurological diseases which correlated with activated microglia (Figure 1).

Akaishi et al. [22] synthesized AB43, a synthetic pyrazole derivative of curcumin, which was found to suppress lipopolysaccharide (LPS)-induced nitric oxide production through the inhibition of NF-κB and p38 MAPK (p38 mitogen-activated protein kinase) pathways in microglia. They demonstrated, for the first time, that AB43 is a potent inhibitor of LPS-induced iNOS expression and NO production in brain microglia (Figure 3).

Chen et al. [42] found that AB52 reduced inflammatory response in huAPP/PS1 mice (APP/PS1 mice contain human transgenes for both APP, Supplementary Data). AB52 highly upregulates also, 5-lipoxygenase (5-LOX) in the huAPP/PS1 mice lower to control levels. Data verified that AB52 is able to modulate the level of inflammatory response in huAPP/PS1 mice (Figure 3). 

**Figure 6:**
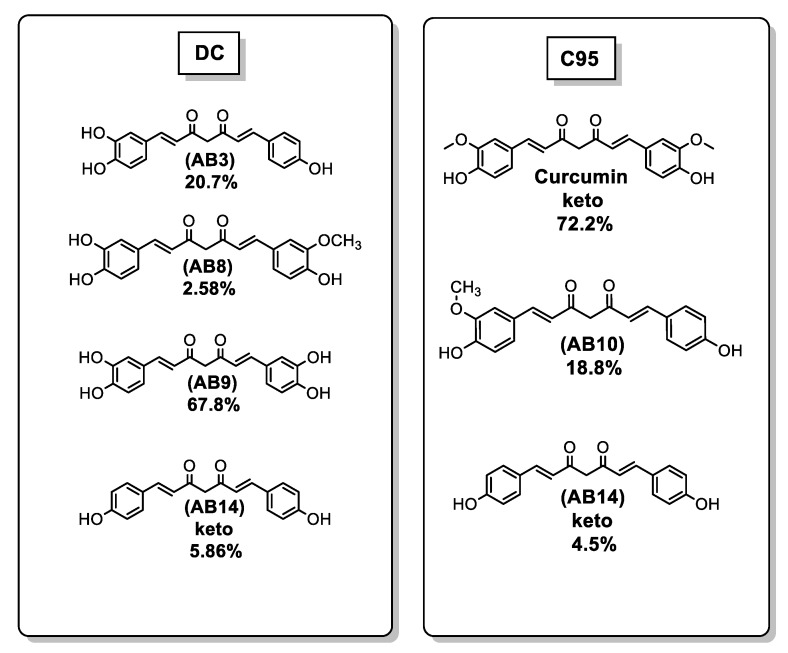
Mixed curcuminoids having antioxidant properties.

It is known that curcuminoids are able to inhibit proinflammatory induction. Specifically, curcuminoids are known to attenuate the proinflammatory effects of TNF-α [21]. Khanna et al. [20] compared the effects of DC (67.8% AB9, 20.7% AB3, 5.86% AB14 keto form, and 2.58% AB8) and C95 (72.2% curcumin, 18.8% AB10, and 4.5% AB14 keto form) in a model of TNF-α–induced gene expression in human microvascular endothelial cells (HMECs, Supplementary Data) (Figure 6).

Gagliardi et al. [80] showed that treatment with curcuminoids, in a cell model PBMC (peripheral blood mononuclear cells, Supplementary Data), inhibited the activation of inflammation. AB14 showed the most potent protective activity decreasing levels of NF-κB and BACE1 and the inflammatory cascade in cells from AD patients (Figure 1).

**Figure 7:**
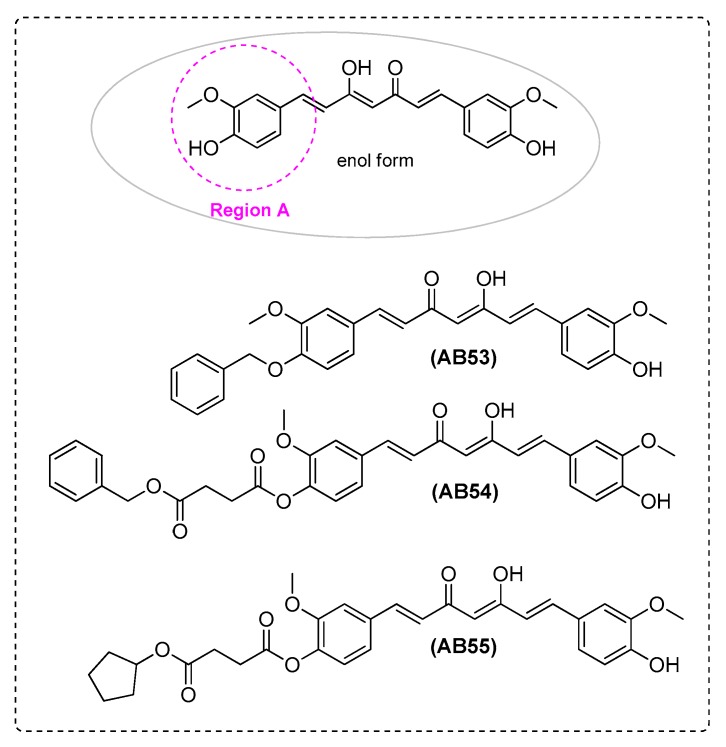
Anti-inflammatory inhibitors.

Lakey-Beitia et al. [7] found novel curcumin derivatives as potent inhibitors of inflammation in AD. AB21 (Figure 1), AB53, AB54, and AB55 (Figure 7) decreased the secretion of IL-6, depending on the chemical modification. AB21 and AB53 downregulated the production of IL-6 in a concentration-dependent manner, with a negligible release at 10 μM. Curcumin derivatives were subjected to a SAR analysis. Changes in the hydroxyl groups located on the aromatic rings were made and the anti-inflammatory activity of the derivatives was evaluated. Etherification of the hydroxyl groups, on both aromatic rings, showed a higher anti-inflammatory activity. 

Furthermore, the introduction of a benzene ring etherified at one of the curcumin rings led to a more potent anti-inflammatory curcumin derivative (AB53). Acetylation, on only one side of the molecule, was associated with potent biological activity. However, the complexity and the spacer’s length attached to both rings decreased the activity. Indeed, a strong improvement in the activity was achieved when modifications were done on only one of the aromatic rings (AB54, AB55). When monofunctionalized diester curcumin derivatives were considered (AB54, AB55), it was observed that the improvement of the anti-inflammatory activity was reduced by the presence of bulky groups in the molecule. These bulky groups disturbed the molecular mechanisms by which these derivatives inhibited the production of IL-6. This observation needs further investigation. The researchers suggested that hydroxyl groups on the aromatic rings of the curcumin were elements of the pharmacophore, prerequisites for reducing the production of IL-6. Moreover, modifications appropriate to produce new analogues with potential anti-inflammatory activity targeting IL-6 includedthe following structural moieties: at least one hydroxyl group of the aromatic rings should not be modified, etherification and esterification of only one of the hydroxyl groups.

Bisceglia et al. [18] examined the anti-inflammatory activity of analogue AB19 and curcumin (Figure 1). In addition, Deck et al. [81] examined enone analogues of curcumin as Nrf2 activators.

**Figure 8:**
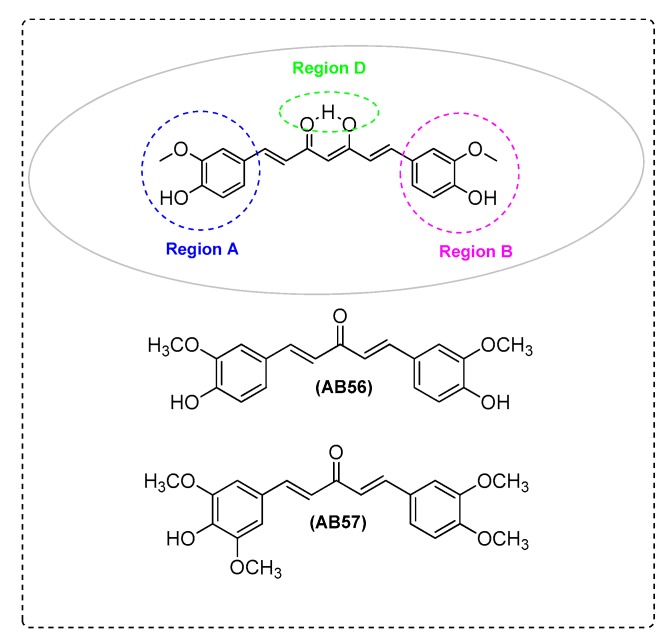
Anti-inflammatory inhibitors.

The transcription factor Nrf2 is a major regulator of phase II detoxification. Nrf2 is located in the CNS and is highly implicated in brain’s inflammation as an important regulator. AB19 (Figure 1) [18], AB56, and AB57 (Figure 8) [82] were also found to be involved in Nrf2 signaling.

### 2.4. Antioxidant Activity 

Oxidative stress is a complicated multifactorial situation. Decreased levels of the brain antioxidant GSH [83] lead to an AD-related increase. GSH is implicated in several actions which include: (i) control of gene expression, (ii) apoptosis, (iii) penetration of membranes, and (iv) detoxification of potentially toxic electrophiles and metals. This latter protects cells from ROS [84].

In addition, in AD, the presence of heme oxygenase 1 (HO-1) protein expression is increased. HO-1 has a protective antioxidant role but its chronic upregulation induces degeneration followedby accumulation of iron and oxidative damage of mitochondria [85].

Research has suggested that mitochondrial dysfunction, metal accumulation, hyperphosphorylated tau, inflammation, and amyloid-β (Aβ) accumulation are the basic mechanisms underlying the induction of oxidative stress [86]. Thus, the reduction of oxidative stress could serve as a target for designing curcumin derivatives.

Chen et al. [42] found that AB52 decreased HO-1 in huAPP/PS1 mice to control levels (Supplementary Data). HO-1 is frequently thought of as an antioxidant enzyme, but it is elevated in AD brain and can act as a pro-oxidant factor under some conditions (Figure 3 and Table 2).

Xu et al. [82] found that a low dose of AB56 and AB57 increasd Nrf2/HO-1 protein expression and decreased Kelch-like ECH-associated protein 1 (Keap1), in PC12 cells. In addition, AB56 and AB57offer cytoprotection against the oxidative damage induced by Aβ_25–35_. Ultimately, the neuroprotective effect of AB56 and AB57 provides a pharmacological basis for their clinical use in prevention and treatment of AD. Low dosages of AB56 and AB57decrease ROS levels, following similar mechanism as with curcumin. The mono-ketone analogues present improved stability, and the phenolic OH is essential for scavenging activity, while instability of curcumin can be ascribed to the β-diketone moiety (Figure 8 and Table 2).

Bisceglia et al. [18] studied the scavenging ability of prenylated curcumin analogues in SH-SY5Y cells (Supplementary Data), when coincubated with H_2_O_2_, using curcumin as reference. Treatment with curcumin and compounds AB19 reduced H_2_O_2_-induced intracellular ROS production. Curcumin showed the highest scavenging activity as compared with analogues AB19. In particular, the presence of vanillin moieties (as for curcumin) appeared to be important for this antioxidant activity. Compounds lacking the 4-hydroxy, 3-methoxy group on both aromatic rings were less effective than compound AB19, in which one of the two vanillin functions of curcumin was preserved. Curcumin and AB19, at a concentration of fiveμM, induced Nrf2 nuclear translocation. The asymmetrical analogue AB19, at a concentration of 10 μM, did not produce significantly statistical experimental results. Nrf2 activation is not the only antioxidant path. AB19 caninfluence the antioxidant activity through different mechanisms. Another important pathway could be miRNA modulation. Further research is in progess to define the antioxidant mechanism of the derivatives (Figure 1 and Table 2). Additionally, Deck et al. [81] examined enone analogues of curcumin on Nrf2 protocol to define Nrf2 activators. The tested compounds were divided into three groups presenting the following: (i) a seven-carbon dienonelinker, (ii) a five-carbon enone linker with and without a ring, and (iii) a three-carbon enone linker. Among the three groups, several activators of Nrf2 were found more activeas compared with curcumin (Table 2).

Xiao et al. [63] indicated that Aβ could induce apoptosis, oxidative stress, and inhibition of telomerase reverse transcriptase (TERT) expression in SK-N-SH cells (neuroblastoma cell line, Supplementary Data). Their study demonstrated protective effects of curcumin and a keto form of AB14 against Aβ neurotoxicity in vitro. Nevertheless, the protective effects of curcumin and keto form of AB14 were lost while telomerase was scarce. In view of the special advantages of curcumin and keto form of AB14 (such as the penetration of blood–brain barrier, BBB), the researchers suggested that curcumin and keto form of AB14 could be used as a potential therapeutic agent for AD. Moreover, data showed that neuroprotective effects of curcumin and keto form of AB14is dependent on telomerase. Thus, telomerase could be a target for the therapeutic effect of curcumin and keto form of AB14. ROS were increased significantly and GSH declined significantly after exposure to Aβ_1–42_ for 24 h. However, a significant decrease in ROS generation and a significant increase in GSH level was observed when a pretreatment with curcumin or keto form of AB14 was followed. Keto form of AB14 was found to be a better antagonist of Aβ_1–42_ -induced oxidative stressas compared with curcumin. Curcumin, as well as keto form of AB14, could reduce oxidative stress. In previous studies, it was found that the 4-hydroxyl group could be responsible for the neuroprotection of curcumin and keto form of AB14 (Figure 1 and Table 2).

Mishra et al. [55] synthesized AB7 which had beneficial effects in AD and other neurodegenerative diseases involving oxidative stress and neuronal loss. They showed that AB7 reducedthe amyloid-β-induced increase in the level of ROS (Figure 1 and Table 2).

Khanna et al. [20] examined the effects of DC (67.8% AB9, 20.7% AB3, 5.86% AB14 keto form, and 2.58% AB8) on the concentration of basal cellular GSH and ROS.DC, at 100 ng/mL, significantly increased cellular GSH; however, the cellular ROS was not affected. At 500 ng/mL, DC was noted to protect HT4 (mouse hippocampal cell line) against glutamate toxicity, and significantly lowered basal cellular ROS levels. These results support the antioxidant properties of DC (Figure 6 and Table 2).

Derivative AB15, palmitic acid curcumin ester, was synthesized and characterized by Qi et al. [60]. This curcuminoid, cultivated on the membranes of neurocytes prevented Aβ-mediated ROS production. Furthermore, AB15 could scavenge in vitro and in vivo Aβ-mediated ROS as curcumin. Morphological analyses showed a better protecting cell shape for AB15. Specifically, AB15 significantly reduced lipid peroxidation in the presence of soluble Aβ. The protective effect of AB15 was lower than curcumin, due to the absence of phenol groups. Similar anti-lipid peroxidation activitycaused by fibrillar Aβ was observed. However, in the presence of copper ions, AB15 was more potent than curcumin (Figure 1 and Table 2). 

**Figure 9:**
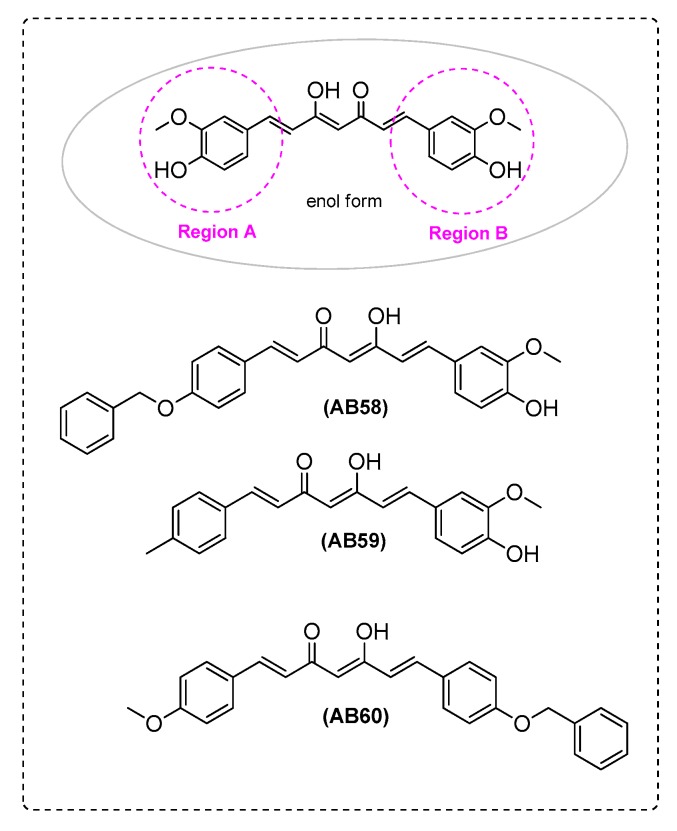
Antioxidant inhibitors.

Di Martino et al. [26] studied the ability of curcumin-based derivatives to protect from oxidative stress on T67 cells (human glioma cell line, Supplementary Data). AB58 showed medium antioxidant, lower as compared withcurcumin. The lack of scavenging activity observed by AB59 suggested that this property could not only be ascribed to the presence of the 3-OCH_3_, 4-OH-aryl function (Figure 9). The research group studied NAD(P)H:quinone acceptor oxidoreductase 1 (NQO1) induction [95]. The tested compounds proved to be NQO1 inducers. In particular, AB17 and AB14 (Figure 1) were as active as curcumin, while higher activities were observed by AB58, AB59, and AB60 (Figure 9 and Table 2).

Fang et al. [16] suggested that the phenolic hydroxy groups clearly play an important role in the antioxidant activity. All the target dimethylaminomethyl-substituted curcumin derivatives, which preserve at least one phenolic hydroxy group, showed positive antioxidant effect. Particularly, compound AB22 which has two phenolic hydroxy groups exhibited higher free radical scavenging activities (FRSA). AB22 showed good stability. Furthermore, AB22 showed good lipophilicity (logP = 3.48), suggesting a potential ability to penetrate the BBB (Figure 1 and Table 2).

Hotsumi et al. [39] found that o-dihydroxy phenolic derivatives are potent radicalscavengers. It should to be noted that the conjugation with a carbonyl group decreases the scavenging ability. In general, the curcumin analogues showed moderate radical scavenging activities (IC_50_ = 10 to 99 μM) (Table 2).

In Kalaycıoğlu et al.’s [23] research the antioxidant activities of curcuminoids were decreased in the order curcumin >AB10>AB14 (Figure 1 and Table 2).

Bukhari et al. [58] found that AB41 exhibited strong FRSA (18.39 μΜ. All strong antioxidant compounds possess protective effect against Aβ-induced PC12 cell death, Supplementary Data (Figure 3 and Table 2).

Akaishi et al. [22] measured the oxygen free radical scavenging capacity of AB43, a synthetic pyrazole derivative of curcumin. AB43 has phenolic rings in its structure supporting its antioxidant activity. The O_2_^•-^ scavenging activity of AB43 was lower than the reference curcumin, however, the OH^•^ scavenging activity of AB43 was similar to that of curcumin (Figure 3 and Table 2).

**Figure 10:**
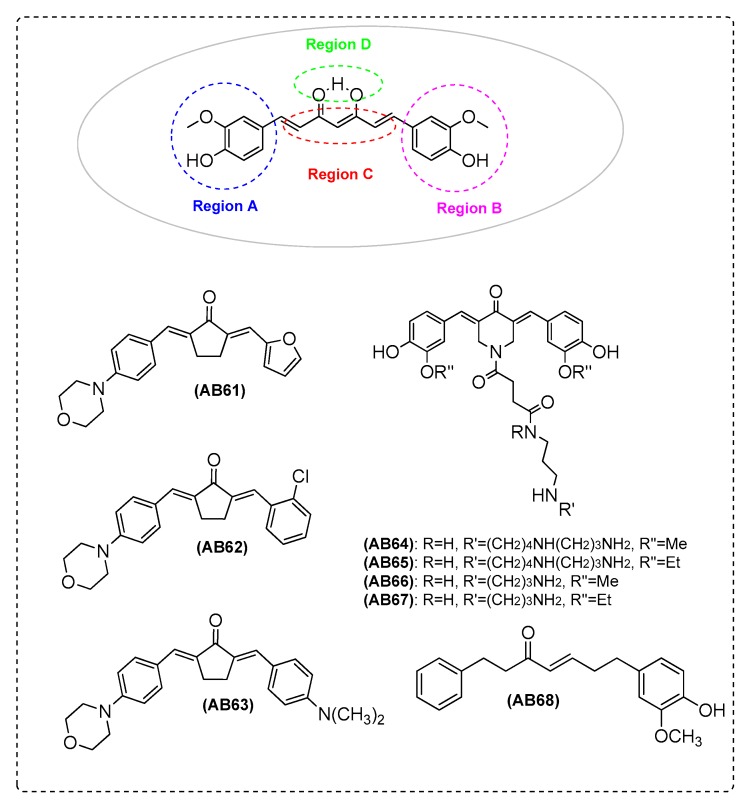
Antioxidant inhibitors.

Furthermore, there are curcumin analogues and derivatives which were evaluated for their antioxidant activity in vitro. Chen et al. [11] tested several compounds using the oxygen radical absorbance capacity assay using fluorescein (ORAC-FL). The absence of phenolic groups (methoxy or hydroxy) affected the antioxidant activity. These moieties are important for sxavenging activity. Among the tested compounds, AB42 presented the highest ORAC value (5.8), much higher than Trolox (1.0) and curcumin (2.5). In addition, the ability of the compounds to counteract the formation of ROS was assayed in SH-SY5Y (Supplementary Data). Further research is in progress to optimize the structure of AB42. These attempts should drive the development of more efficient multitarget anti-Alzheimer agents (Figure 3 and Table 2). Additionally, Zhai et al. [9] analyzed the data of ORAC. Compounds with groups such as methoxyl or hydroxyl were important for the antioxidation activity of curcumin and exhibited better antioxidation activity than Trolox. However, it was found that compounds AB61, AB62 (Figure 10), and AB47 (Figure 3) without the abovementioned groups also exhibited antioxidant activity. It is possible that the styryl function and steric or electronic factors through the large aromatic structure contributed to the antioxidant activity. AB46 and AB48 (Figure 3) also showed excellent antioxidative activity. The ORAC-FL results from AB63 (Figure 10) (3.1) preserved a dimethylamino group on the benzene ring, showing excellent scavenging activity (Table 2).

#### 2.4.1. Mitochondrial Dysfunction and Apoptosis

Oxidative stress expresses the imbalance between ROS and detoxification procedures leading to disorders in mitochondria functions, as well as cell apoptosis. During the apoptosis, mitochondrial integrity and the release of mitochondria-initiated factors are maintained inB-cell lymphoma 2 (Bcl-2) family members. Bcl-2-like protein 4 (Bax) through the induction ofROS production and mitochondria depolarization, could enhance the release of cytochrome complex (Cyt-c) into cytoplasm, activating downstream caspase, leading to cell apoptosis [82].

Simoni et al. [8] synthesized polyamine conjugated curcumin analogues as antioxidants by means of indirect antioxidant mechanisms such as HO-1 induction. These compounds are efficient pleiotropic antioxidativescapable of penetrating cells mebranes and being inside the mitochondria. Polyamines that take advantage of electrostatic forces have been identified as tools for a transport system into mitochondria. Polyamines could also fine tune the bioactivity of a compound. It should be noted that the combination of a polyamine moietywith the curcumin-like scaffold leads to a new entity that is able to target mitochondria and present efficient intracellular uptake. The compounds resulted in a significant decrease in the cytotoxicity effects. AB64, AB65, AB66, and AB67 are promising leads for neuroprotection lead discovery. At a dose of 10 μM, AB64, AB65, AB66, and AB67 significantly decreased ROS production, similar to curcumin (Figure 10 and Table 2).

Molecular dynamic (MD) simulation and molecular docking studies performed by Iwuchukwu et al. [88] showed that AB52 is bound to the allosteric α subunit (ATP5A) of the target protein, which is a mitochondrial adenosine triphosphate synthase (mATP synthase) and alters its biological activity, which basically involves ATP hydrolysis and synthesis. This ability of AB52 underlies itsusefulness as a therapeutic agent in the treatment of AD and helps the design of new modulators of mATP synthase based on a structural analysis (Figure 3 and Table 2).

Shi et al. [89] investigated the neuroprotective effects of curcumin analogue, AB68, on oxygen-glucose deprivation and reoxygenation (OGD/R) induced injury in cortical neurons. AB68 increases the resistance of the cortical neurons to OGD/R (Figure 10 and Table 2).

AB7 was found [91] to protect neurons from traumatic brain injury (TBI) induced apoptotic neuronal death.AB7 reduces amyloid-β-induced increase in the level of reactive oxygen species, the decrease in mitochondrial membrane potential, and the caspase activation [55]. Thus, AB7 is an attractive therapeutic agent for TBI, and therefore for neurological diseases such as AD (Figure 1 and Table 2).

**Figure 11:**
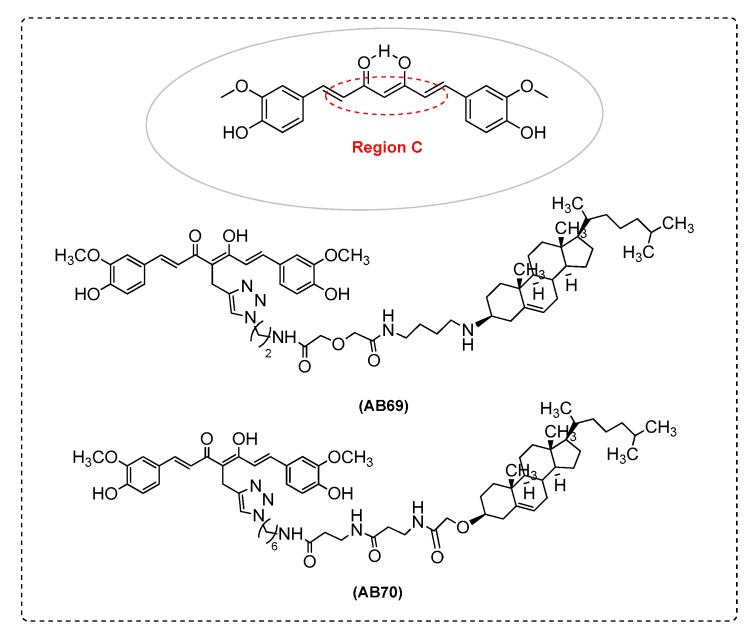
Antioxidant inhibitors.

Liu et al.’s [90] research in a cellular AD model with bivalent compounds found that theyoffer high neuroprotection as multifunctional agents. MC65 cells (Supplementary Data) exhibit increased mitochondrial membrane potential and bivalent compound AB69 can reverse this increase. It is known thatcytosolic Ca^2+^ is increased upon tetracycline (TC) removal in MC65 cells and AB69 can abolish this change. Bivalent compound AB69 can readily pass into MC65 cells and colocalize with mitochondria and ER which suggests a multiple-site mechanism (Figure 11 and Table 2).

Saathoff et al. [92] examined compound AB70 in MC65 cells (Supplementary Data). Results demonstrated that AB70 suppressed the change of matrix metallopeptidase (MMP) reacting with the mitochondrial complex I in MC65 cells. It seems that bivalent compounds with varied linker length and an anchor to hook to the cell membrane can exhibit a different mode of neuroprotection (Figure 11 and Table 2).

Xu et al. [82] found that compounds AB56 and AB57 could increase Bcl-2 expression level and decrease the level of Bax and Cyt-c in Aβ_25–35_-treated PC12 cells (Supplementary Data). These observations support the clinical studies for prevention and treatment of AD (Figure 8 and Table 2). 

Another curcumin analogue was found by Pinkaew et al. [6] that is involved in apoptosis, which is AB9. AB9 was found to increase the ratio of B-cell lymphoma-extra large (Bcl-XL)/Bax protein, to reduce intracellular ROS level and cytochrome c protein expression, and to cleave caspase-9 (cysteine-aspartic protease 9) protein expression as well as caspase-3 protein expression. It was suggested that AB9 could protect from neuronal death suppressing the apoptosis mediated by mitochondrial death and ER stress pathway (Figure 1 and Table 2).

**Figure 12:**
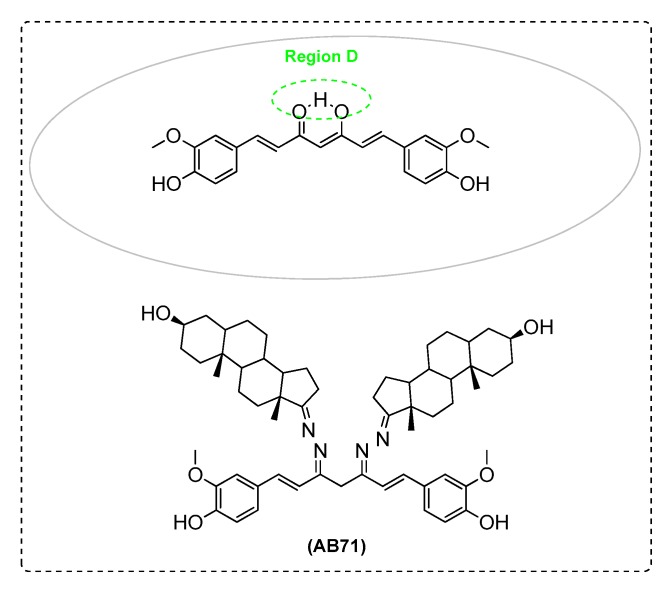
Antioxidant inhibitor.

**Figure 13:**
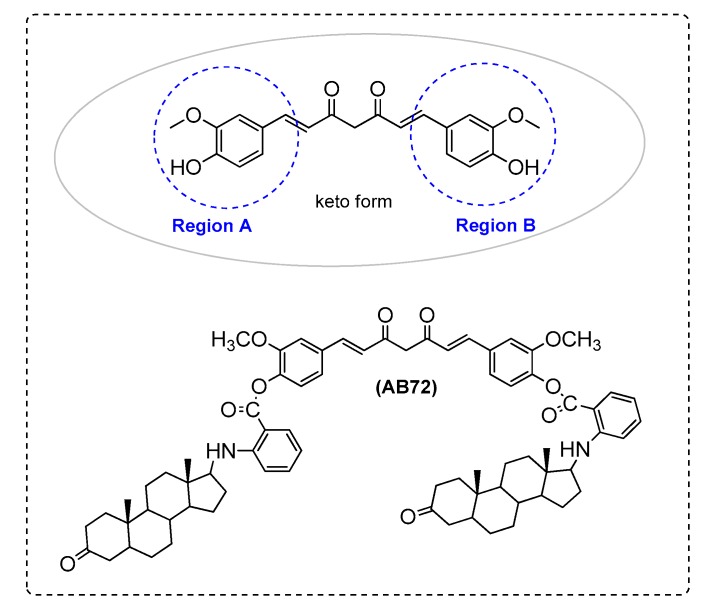
Antioxidant inhibitor.

**Figure 14:**
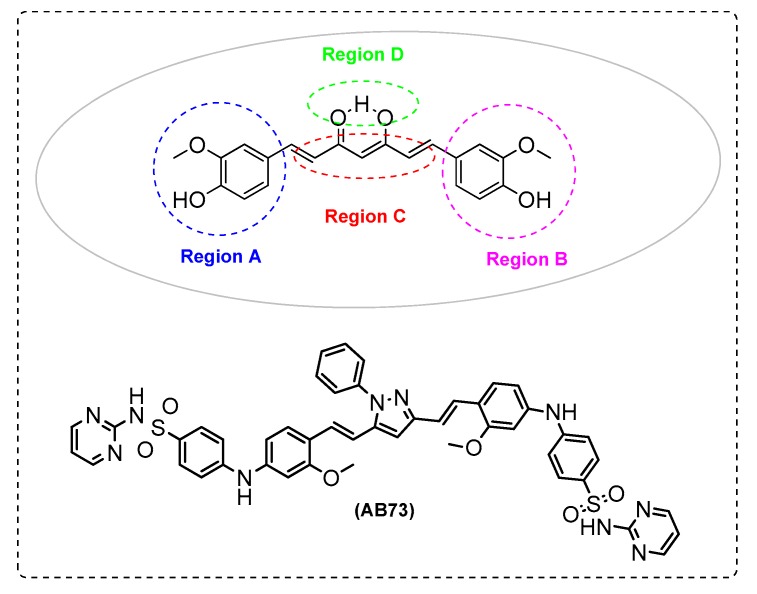
Antioxidant inhibitor.

Elmegeed et al. [87] synthesized novel curcumin analogues containing promising heterocyclic nucleus fused to the essential pharmacophore feature of the curcumin moiety. Specifically, the aim of this study was extended to elucidate the efficacy of these novel synthesized compounds in the reduction of AD induced in adult female albino rats. The results revealed that treatment of AD groups with compounds AB71 (Figure 12), AB72 (Figure 13), AB73 (Figure 14), or rivastigmine significantly increased GSH, paraoxenase, and Bcl2 levels in brain. The FRSA of the tested compounds correlated with the phenolic OH group (AB71), the CH_2_ group of the β-diketone moiety (AB72), and with the pyrazole ring and the methoxy groups (AB73). Moreover, the presence of the steroid moiety in compounds AB71 and AB72 was found to enhance theirpreventive role against neurodegenerative disorders (Table 2).

#### 2.4.2. Metal Accumulation and Metal Ion Dyshomeostasis

Abnormal enrichment of Cu^2+^, Fe^2+^, and Zn^2+^ in post-mortem AD brains has been observed. These metal ions present high affinity for Aβ and induce the formation of Aβ aggregates. Neurotoxicity is significantly connected with the combined presence of metals and Aβ [96,97].

Chen et al. [11] found that the compound AB42chelated metals such as iron and copper and decreased metal-induced Aβ aggregation (Figure 3 and Table 2).

Ferrari et al. [27] synthesized curcumin derivatives which had the ability to bind Cu(II) ion and Ga(III) ion. Metal ion homeostasis is tightly regulated to maintain physiological processes. Thus, imbalances of metal ion concentrations, especially of Cu^2+^, can favor Aβ aggregation and, subsequently, induce oxidative damage. The experimental ^1^H and ^13^C chemical shifts and the calculated Gauge-Independent Atomic Orbital (GIAO) were found to have a good correlation. Furthermore, the calculated BDE (O-H bond dissociation energy), O-H proton dissociation enthalpy (PDE), calculated electronic affinity (EA), and ionization potential (IP) values for AB31 and its complexes were compared to the corresponding curcumin, predicting the antioxidant properties of metal complexes. The BDE values of the ligands were compared to previously reported antioxidant activity against 2,2-diphenyl-1-picrylhydrazyl radical scavenging (DPPH), suggesting that charge delocalization plays an important role in determining the scavenging ability. Therefore, the complexes of gallium(III) look very promising as superoxidescavengers suggesting their possible use as SOD mimics (Figure 2 and Table 2). Additional research [40] revealed that the co-ordination of copper ion by the keto-enolic group has a protecting role since the involvement of the keto-enolic moiety in the radical delocalizationis prevented. Among the different proposed reaction mechanisms, the involvement of the keto-enolic group in stabilizing the formed radical through delocalization was also reported. The use of copper(II) chelating agents can reduce the damage induced by the free metal ion. The antiradical activity of copper complexes showed that only AB31 scavenges DPPH radical, describing the importance of a phenolic group in combination with methoxyl moiety in ortho position (Figure 2 and Table 2).

Zhai et al. [9] suggested that AB48-metal(II) complex is formed in solution. Similar behavior was also observed with Fe^2+^ and Zn^2+^. AB48 could be useful in the treatment of AD acting as as metal chelator (Figure 3 and Table 2).

Kochi et al. [29] studied the inhibitory activity of curcumin derivatives towards metal-free and metal-induced amyloid-β aggregation. It seems that AB26 could highlydecrease Cu(II)-triggered Aβ aggregation. In addition, the promising increase in solubility of AB26 in aqueous media and its reactivity towards metal-free Aβ and metal-Aβ species in vitro, demonstrates that structural modifications of curcumin are worthy for improvement of its properties. These results are promising for the development of potential theranostic agents (both diagnostic and therapeutic) for neurodegenerative diseases (Figure 1 and Table 2).

The bivalent ligand AB69, with its spacer (length of 17 atoms) formed a complex with biometals, such as Cu, Fe, and Zn to provide new analogues with novel pharmacological activity and potency (Figure 11 and Table 2) [62].

### 2.5. Inhibition of AChE

Until now, AChE inhibitors (AChEI) were the major class of drugs approved for AD, providing symptomatic relief and improvement in cognitive function. Researchers are tryingto synthesize innovative curcumin-based drugs that would have better pharmacokinetic properties, as well as increased affinity to the target.

Ahmed et al. [98,99] used in vitro and ex vivo models of AChE inhibitory activity to study the effect on rat memory. Curcuminoids (a mixture of curcumin, AB14 and AB10) inhibited AChE in the in vitro assay with a IC_50_ value of 19.67, AB14 16.84, AB10 33.14, and curcumin 67.69 μM. All compounds, at a fixed dose (10 mg/kg), showeda significant (*p*<0.001) and comparable effect in scopolamine-induced amnesia. The ex vivo experiments showed a dose-dependent (3 to 10 mg/kg) inhibition. These data indicate the anti-AChE activityof all the tested compounds, with the exception of curcumin (Figure 1).

**Figure 15:**
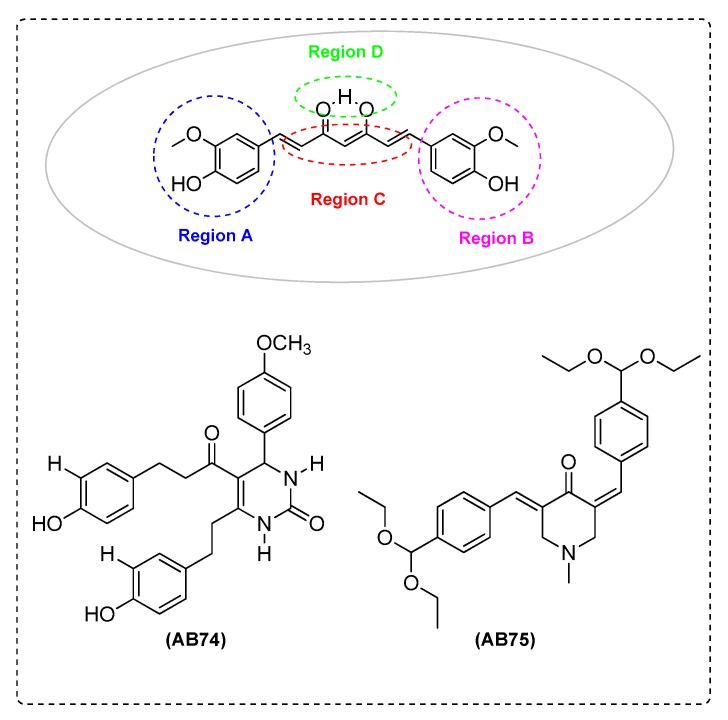
AChE inhibitors.

Arunkhamkaew et al. [100] synthesized AB74 analogue bearing a 4-OCH_3_ phenyl group and evaluated as an inhibitor of AChE. AB74 was found to be a potent inhibitor of AChE with an excellent IC_50_ value of 1.34 ±0.03 μM, which is slightly higher than that of galanthamine (Figure 15).

Tello-Franco et al. [101] carried out a computational approach to dock the curcumin analogues to the active site of AChE. They analyzed the hydrogen bonding and the interactions to relevant aromatic amino acids. They tried to define the common structural features between the known AChEIs and the tested derivatives. They suggested thattwo aromatic rings and the appropriate distance between them were the prerequisitesfor a favorable interaction of curcumin and its derivatives with both the quaternary and peripheral sites of AChE. The complexis stabilized by hydrogen bonds with the quaternary and acyl sites. The inhibitory activity was decreased by the acylation of the hydroxy groups and the reduction of the conjugated double bonds. Thus, the modification of the keto-enol moiety is characterized as the best alternative for the design of more potent AChE inhibitors.

Bukhari et al. [58] found that compounds AB75 (Figure 15) and AB41 (Figure 3), containing *N*-methyl-4-piperidone linker, showed high AChE inhibition as compared with the reference drug donepezil. It was also observed that the most potent AChE inhibitors within the series present the *N*-methyl-4-piperidone and 4-piperidone moieties. The nature of the aromatic substituent does not influence inhibitory activity. 

The researchers performed molecular docking and QSAR studies to determine the structural features and the physicochemical properties that are responsible for the AChE inhibition activity.

For QSAR analysis, a great number of descriptors were calculated followed by elimination of redundant descriptors using QSARINS software (Supplementary Data). A GA-MLR (genetic algorithm multilinear regression) was performed to develop robust QSAR model. The best two parameters of QSAR follows:

pIC_50_ =126.3811 (±28.0863) − 7.2606 (±2.3866) RDF090m − 4.8896 (±2.0119) F01[C-N].

N_tr_ = 14, N_ex_ = 3, R^2^ = 0.8558, R^2^_adj_ = 0.8296, CCC_tr_ = 0.9223, F = 32.6359, Q^2^LOO = 0.7924, CCC_cv_ = 0.8897, R^2^_ex_ = 0.7718, CCC_ex_ = 0.8578.

The high value of the square of correlation coefficient (R^2^), the adjusted determination coefficient (R^2^_adj_), the concordance correlation coefficient of the training set (CCC_tr_), Fisher ratio (F), the square correlation coefficient for leave-one-out cross-validation (Q^2^LOO), test set using LOO cross-validation (CCC_cv_) and coefficient of determination (R^2^_ex_) indicates that the QSAR model is robust offering good predictive ability. N_tr_ and N_ex_ represent the number of samples in the training set and external set, respectively.

From the QSAR model, it is clear that activity is correlated with RDF090m (radial distribution function 9.0/weighted by atomic masses, RDF descriptors) and F01[C-N] (frequency of C-N at topological distance of 01, a 2D frequency fingerprints descriptor).

Elmegeed et al. [87] found that the steroidal curcumin derivativesAB71 (Figure 12), AB72 (Figure 13), and AB73 (Figure 14) decreased AChE activity in brain.

Kalaycıoğlu et al. [23] studied AChE and BChE inhibitory activities of tested curcuminoids (curcumin, AB10, and AB14). AB14 showed significant AChE inhibition as compared with galantamine. Curcumin exhibited lower activity as AChE inhibitor. Furthermore, curcumin and AB10 did not inhibit BChE. On the contrary, AB14 presented inhibition of BChE (Figure 1).

Wilar et al. [102] found that curcumin, AB10, and AB14 significantly suppressed the priming effects of nicotine and inhibited AChE activity (Figure 1).

## 3. Curcumin Hybrids for AD Therapy

Development of MTDLs has emerged as a promising approach to target the complex etiology of AD. Donepezil, an AChEI, is a known anti-AD drug. Although it presents symptomatic efficacy, its effecton the AD process are still under investigation.

**Figure 16:**
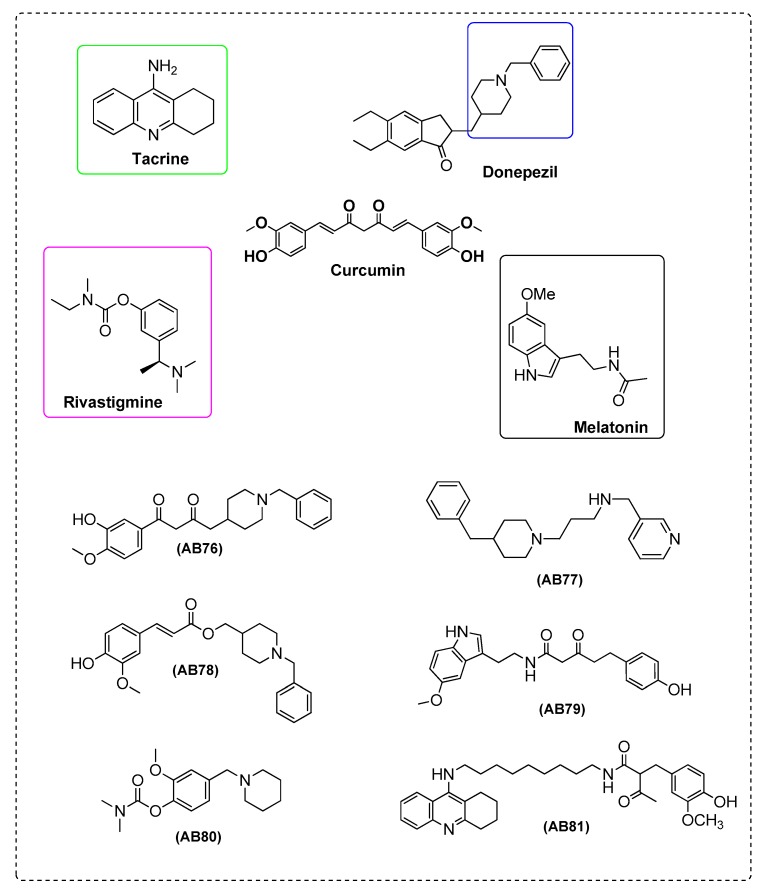
Curcumin-based hybrids.

Yan et al. [24] designed, synthesized, and evaluated a series of novel curcumin derivatives as MTDLs for the treatment of AD, by fusing donepezil and curcumin. Thus, they tested as inhibitors of AChE, of BChE, of Aβ self-aggregation, as antioxidants and as metal chelators. AB76 seems to be the best derivative, since it enhancedthe cholinergic function in the CNS and displayed high ant-BChE/anti-AchE selectivity. In addition, in vitro, AB76 inhibited self-induced Aβ aggregation, effectively penetrated the BBB, and presented antioxidant activities (Figure 16).

Meena et al. [68] designed and synthesized a new series of *N*′-(4-benzylpiperidin-/piperazin-/benzhydrylpiperazin-1-yl)alkylamine derivatives which they biologically evaluated as inhibitors of AChE, amyloidbeta (Aβ) self aggregation, and for their radical scavenging activity. The design of the compounds was based on curcumin and donepezil. The in vitro studies showed that almost all the synthesized compounds were potent inhibitors of AChE and BuChE with nanomolar IC_50_ values and more potencyas compared with the known drug donepezil. CompoundAB77presented high selectivityfor AChE (~38-fold than donepezil, IC_50_ = 2.13 nM), strongly inhibiting AChE. Docking analysisconfirmed that AB77is bound simultaneously to the catalytic active site and the peripheral anionic site of AChE. Electron rich substituents on the aromatic ring and a benzylpiperidine scaffold influenced positively the inhibition of Aβ_1–42_ self-aggregation. The derivatives containing methoxy and hydroxy groups showed potent ORAC ranging from 2.2- to 4.4-fold of the Trolox value. All compounds present druglikeness (Figure 16).

Morroni et al. [69] studied a novel feruloyl–donepezil hybrid compound AB78. The researchers prepared this compound and tested it as a multitarget agentfor the treatmentof neurotoxicity induced by the administration of Aβ_1–42_ oligomer (AβO) in mice. Ferulic acid is produced by the degradation of curcumin.(Figure 16) [93]. AB78 is known to exert anti-inflammatory activity in different in vivo models and neuroprotective activity in human neuronal cells. AB78 (0.5 to 1 mg/kg) reduced oxidative damage and neuroinflammation Moreover, AB78 increased brain plasticity and protected mice against the decline in spatial cognition. AB78 modulated different pathways as compared with donepezil and it was found more effective in counteracting AβO damage (Figure 16).

Chojnacki et al. [65] designed and synthesized novel compounds asneuroprotectors by hybridization of curcumin and melatonin. The lead hybrid AB79showed significant neuroprotection in the nanomolar range (EC_50_ = 27.60 ± 9.4 nM) in MC65 cells. Multiple in vitro assays established thatAB79 exhibited moderate inhibition on the production of AβOs in MC65 cells (Supplementary Data), but not on the aggregation of Aβ species. It also exhibited significant antioxidative properties. Furthermore, AB79, after oral administration, is biodistributed to the brain (Figure 16). In addition, Gerenu et al. [66] reported that AB79lowered te levels of Aβ in the hippocampus and cortex regionand decreased Aβ burden in APP/PS1 mice (Supplementary Data) after long-term treatment as well. AB79 reduced inflammatory responses and oxidative stress. Furthermore, AB79 significantly improved synaptic dysfunction, indicating its protective effects on synaptic degeneration (Figure 16).

Li et al. [103] based on rivastigmine and curcumin, designed, synthesized, and evaluated a series of novel 2-methoxy-phenyl dimethyl-carbamate derivatives as MTDLs. The results were promising with sub-micromolar IC_50_ values for AChE and BChE inhibition.AB80showed the highest anti-AChE inhibitory activity (IC_50_ = 0.097 μM, 20-fold higher than that of rivastigmine). In addition, AB80 demonstrated inhibitory activity against Aβ self-aggregation similar to cucurmin, whereas rivastigmine presents a weak activity. Moreover, the hydrolysate of AB80showed potent ABTS^+^ scavenging and moderate copper ion chelating activity in vitro. Judging the structural characteristics of AB80 it seems that ortho-methoxy carbamate moiety can play an important role in binding to Aβ_1–40_ (Figure 16).

Liu et al. [94] designed and synthesized tacrine-curcumin hybrid compounds for multifunctional anti-Alzheimer’s agents. In vitro studies showed that these hybrid compounds exert good AChE inhibitory activity, especially AB81. Some of the compounds in correlation to their structure exhibited different selectivity on AChE or BChE. These hybrid compounds possessed pronounced antioxidant activity and effectively protected PC12 cells from the H_2_O_2_/Aβ_42_-induced toxicity. They also showed in vitropositive metal ionschelating ability. All findings demonstrated that the tacrine-curcumin hybrid compound, AB81, can be considered as a potent therapeutic agent for AD (Figure 16).

## 4. Curcumin-Based Imaging Probes for Alzheimer’s Therapy and Diagnosis 

The in vivo diagnosis of AD is of high social and economic impact and remains a demanding field of research. Diagnostic imaging always plays an important role in the management of AD. Magnetic resonance imaging (MRI) is a helpful diagnostic tool in AD. However, it is not helpful due to the changes that happen in human brain volume by aging. Thus, positron emission tomography (PET) supports a better and more accurate diagnosis [104].

It has been reported that curcumin analogues are bound to Aβ plaques, aggregates, dimers, and monomers, but they cannot bind to other amyloid peptides such as amylin [105]. Researchers have shown that curcumin and its analogues could be bound to both soluble and insoluble Aβs [106]. Notably, to date, scaffolds that are bound to soluble Aβs have rarely been reported. The curcumin scaffold is a unique structure for a second generation Aβ PET tracer development [107]. Therefore, the achievement of a curcumin-based compound, with theranostic properties in AD, would be a very useful drugtool. 

### 4.1. Imaging Probes for Amyloid-β Plaques Detection

In the past decade, an attempt has been made to design and synthesize novel curcumin-based amyloid radiotracers for PET imaging.

Near infrared imaging (NIR) is an attractive tool for early AD detection because of its acceptable depth penetration, noninvasive operation, and inexpensive instrumentation. Although NIR imaging, so far, has been limited to animal studies, some NIR probes could be easily modified to PET imaging probes (Table 3) [108].

Ran et al. [108] tried to design a new probe to meet the requirements of a NIR probe for detecting Aβ deposits noninvasively in vivo. Thus, they synthesized and tested a new NIR Aβ plaque-specific fluorescent probe, CRANAD-2, a difluoroborate diketone representative for cell, tissue, and in vivo imaging in small animals. In vivodetermination of the feasibility of the probe for longitude monitoring of low molecular weight Aβ species (such as oligomers, prefibrilar and fibrils) is currently in progress. Since CRANAD-2 enters the brain and is bound specific to amyloid plaques, a radiolabeled version would be suitable for PET imaging. Furthermore, CRANAD-2could be simultaneously useful as a therapeutic for AD (Table 3).

Zhang et al. [33] indicated that CRANAD-3 is suitable for monitoring short-term and chronic treatments, as well as early molecular pathology. To verify the feasibility of CRANAD-3 for monitoring therapy, they used the fast Aβ-lowering drug LY2811376, a well-characterized BACE-1 inhibitor, to treat APP/PS1 mice (Supplementary Data). Imaging data showed that CRANAD-3 could monitor the decrease in Aβs after drug treatment. In order to validate the imaging ability of CRANAD-3, they used it to monitor the therapeutic effect ofCRANAD-17, a curcumin analogue inhibitor of Aβ crosslinking (Table 3).

It is well known that covalent crosslinking of Aβ could be initialized by the coordination of copper with imidazoles on histidine-13 and 14 (H13, H14) of Aβ peptides [109].

Zhang et al. [34], in their study, found that CRANAD-17 can take the role of an anchor to usher the designed derivative close to H13 and H14 of Aβ. Imidazole groups were involved to antagonize with H13/H14 for copper binding. The observations indicated that CRANAD-17 is capable of inhibiting Aβ_42_ crosslinking induced by copper. This raises the potential for CRANAD-17 to be considered as a diagnostic and also a therapeutic tool (Table 3).

Zhang et al. [122] synthesized a novel bifunctional curcumin analogue CRANAD-28. In vivo two-photon imaging studies suggested that CRANAD-28 could penetrate the BBB and label plaques and cerebral amyloid angiopathies (CAAs). This imaging probe could inhibit the crosslinking of amyloid beta, induced either by copper or by natural conditions. Additionally, they suggested that the fluorescent dyes that present longer emissions, like CRANAD-28, can be used for sequential multi-color labeling to avoid ex vivo section staining. Furthermore, CRANAD-28 is capable of attenuate crosslinking of Aβ_42_ induced by metal ions and natural conditions. These findings support its future use in AD diagnosis and therapy (Table 3).

Sato et al. [123] developed of new curcumin analogue, Me-CUR 9, as fluorescent switchable probe to detect amyloid-β fibrils. It showed excellent fluorescence in the presence of amyloid-β fibrils. The Me-CURs presenting a C7-diketone moiety and phenolic rings substituted with 3,4methoxy groups, showed high fluorescence. Detailed spectroscopic studies indicated that Me-CUR 9 had high molecular planarity, and thus it could strongly bind to the Aβ fibrils. Me-CUR 9 is a fluorescent switchable probe capable of detecting amyloid-β fibrils with high sensitivity. These results support that Me-CUR 9 can become a useful fluorescence probe for diagnosis of AD. Docking studies further support the fact that the localization site of Me-CUR 9 is sustained by hydrophobic interaction and hydrogen bonding networks (Table 3).

Mourtas et al. [67] tried to formulate multifunctional nanosized liposomes to target amyloid deposits in AD brains. A lipid-PEG (polyethylene glycol) curcumin derivative was synthesized and characterized with NIRF. The multifunctional liposomeswere prepared by a curcumin derivative in which, additionally, wasinserted an anti-Transferin antibody as a BBB transport mediator. These productswere characterized, and the fluorescence intensity was measured and found to be increased. The enhancement was noticed when the curcumin scaffold was as a salt of diisopropylethylamine (DIPEA). Both curcumin-derivative liposomes and curcumin-derivative anti-TrF liposomes showed high affinity for the amyloid deposits, on post-mortem brains samples of AD patients. The presence of the anti-TrFhighly enhanced and improved the penetration of the BBB cellular model. The findings support that the presence of an antibody on the curcumin-liposome surface does not target deposit staining, while the presence of the curcumin PEG-lipid conjugate does not decrease their ability to target alyloid deposits in brain. These findings support the potential of such multifunctional nanoliposomes (NLs) for application in AD treatment and diagnosis (Table 3).

Liu et al. [110] developed bivalent ligand BMAOI 14 and tested it as a fluorescent probe capable to be used in the detection of aggregated amyloid-β (Aβ) peptide. To be successful this probe is consisted by a Aβ recognition moiety (this is the role of curcumin) and an anchor to be hooked into the lipid part of the neuronal cell membrane (this role is played by cholesterol). The results show that BMAOI 14 is bound to the monomers, oligomers, and fibrils of Aβ_42._ Since the binding affinities were found to be within the low micromolar to submicromolar range, it seems that BMAOI 14 is a potent Aβ imaging agent candidate. This chemical probe presents many optical properties for use in imaging and can rapidly cross the BBB in vivo. Furthermore, BMAOI14 is specifically bound to Aβ plaques. Further research is in progress to optimizethe BMAOI 14(Table 3).

Several studies reference in curcumin analogues that have been labeled with fluorine-18 [4] and gallium-68 [113,114] for future application in PET imaging, and proton-3 [112], and iodine-125 [111] for future application in SPECT and in MRI imaging, respectively.

Gan et al. [111] studied curcumin analogues in order to detect amyloid-β (Aβ) plaques in the brain. Some compounds showed high binding affinities with Aβ plaques. Fluorescent staining was indicated by 1,5-diphenyl-1,4-pentadien-3-onederivative. The radioiodinated ligand [^125^I]1,5-diphenyl-1,4-pentadien-3-one exhibited high brain uptake and favorable clearance from there. Autoradiography in vitro further confirmed the high affinities of [^125^I]1,5-diphenyl-1,4-pentadien-3-one. The results strongly suggested that [^125^I]1,5-diphenyl-1,4-pentadien-3-onecould be developed for amyloid imaging agent to detect senile plaques in AD (Table 3).

Veldman et al. [112] used autoradiography and found that AB14 presented highest affinity for Aβ containing plaques in cortical AD brain tissue. Subsequently, [^3^H] AB14 showed significantly high specific binding in cortical AD brain tissue as compared with control subjects. These findings support the use of curcumin analogues, especially AB14, as potential radioligands for Aβ plaque neuroimaging (Table 3).

Rubagotti et al. [113] described the first gallium-68 labelled compounds potentially directed to the diagnosis of AD. Findings showed that the curcuminoid complexes ^68^Ga(CUR)^2+^, ^68^Ga(DAC)^2+^ are highly bound to both amyloid fibrils and plaques in vitro, whereas ^68^Ga(bDHC)**^2+^** have a moderate profile. None of the complexes was successful in vivo to detect the amyloid aggregates. However, the use of a radionuclide such as gallium-68 leads to the design of curcumin-like structures as PET radiotracers (Table 3).

Asti et al. [114] synthesized Ga-curcuminoid complexes, namely ^68^Ga-(CUR)^2+^, ^68^Ga(DAC)^2+^, and ^68^Ga(bDHC)^2+^. All the compounds showed high stability in saline human serum, when challenged with diethylenetriaminepentaacetic acid (DTPA) or with Fe^3+^, Zn^2+^, and Cu^2+^ for transchelation or transmetalation studies. The results obtained by ^68^Ga(CUR)^2+^and ^68^Ga(DAC)**^2+^** are especially significant. Both compounds presented high affinities. It seems that the intrinsic fluorescent emission of the Ga-curcuminoid complexes introduces the possibility of synthesizing a mixed radioactive/fluorescent pharmacophore which could be exploited as a dual-mode imaging tool (Table 3).

Lee et al. [4] synthesized and evaluated fluorine-substituted 4,4′-bis-substituted or pegylated curcumin derivatives. Their binding affinities for Aβ_1–42_aggregates were measured and 1-(4-fluoroethyl)-7-(4′-methyl) curcumin 1 had the highest binding affinity (K_i_ = 2.12 nM) [4]. Fluorescence staining of Tg APP/PS-1 mouse brain sections (transgenic mouse (Tg2576), Supplementary Data) demonstrated high and specific labeling of Aβ plaques by 1-(4-fluoroethyl)-7-(4′-methyl) curcumin 1 in the cortex region, which was confirmed with thioflavin-S staining. Radioligand [^18^F]1-(4-fluoroethyl)-7-(4′-methyl)curcumin 1was found to present a suitable partition coefficient (logPo/w = 2.40), and it distributes in normal mice presented improvement in brain permeability (1.44% ID/g at 2 min postinjection) as compared with that of [^18^F]FP-curcumin by a factor of 2.8 and fast wash-out from mouse brains (0.45% ID/g at 30 min post-injection). These findings show that [^18^F]1-(4-fluoroethyl)-7-(4′-methyl)curcumin1could be a promising PET radioligand for Aβ plaque imaging (Table 3).

Kim et al. [115] selected 4′-dimethylamino-4″-(2-(2-fluoroethoxy)ethoxy) curcuminoid for radiolabeling, in vitro and in vivo. Although the ligand was able to distinctively stain Aβ plaques in transgenic mouse brain sections and presented suitable lipophilicity, the in vivo studies of [^18^F]4′-dimethylamino-4″-(2-(2-fluoroethoxy)ethoxy) curcuminoid did not presentsatisfactory brain pharmacokinetics in normal mice. The polar radioactive products taken from [^18^F] 4′-dimethylamino-4″-(2-(2-fluoroethoxy)ethoxy) curcuminoid need to be identified. However, the results could serve as a starting point for the design of metabolically stable ^18^F-labeled difluoroboron-curcumin derivatives useful in Aβ imaging (Table 3).

Cui et al. [116] synthesized a number of variously substituted newdibenzylideneacetonederivatives, for Aβ imaging agents. Most of them presented excellent affinity for Aβ aggregates. The presence of an ortho substituent reduced or abolished the binding. However, parasubstitution was highly tolerant of steric bulk substituents. This leads to the development of novel, easily labeled radioligands for imaging Aβ plaques in vivo. Additionally, the radiolabeled probes with [^125^I] and [^18^F], using autoradiography, exhibited good penetration and fast washout in the mouse brain. A specific plaque-labeling signal was clearly taken for these probes in Tg mouse brain sections (transgenic mouse (Tg2576), Supplementary Data) as well as post-mortem AD brain sections. Taken together, the presented findings highlightthat these novel dibenzylideneacetones can be used as suitable diagnostic tools for AD. More structural changes on the dibenzylideneacetone moiety can lead to more successfulAβ imaging agents for both PET and SPECT (Table 3).

There are promising studies in which the curcumin analogues were, first, successfully labeled with [^18^F] [107,118] and [^19^F] [117] and, then, characterized by using PET or MRI, respectively.

Yanagisawa et al. [117] found that injection of FMeC1in the brain of Tg2576 mice displayed remarkable levels of ^19^F signal. In addition, FMeC1 exhibited an affinity for senile plaques in human brain sections. These findings underlined the use of FMeC1 as a potential ^19^F MRI probe to detectin the brain the amyloid deposition (Table 3).

Rokka et al. [118] successfully synthesized [^18^F]curcumin derivate [^18^F] 2-[3,5-bis (4-hydroxy-3-methoxystyryl)-1Hpyrazol-1-yl]-*N*-[1-[2-(2-(2-fluoroethoxy)ethoxy)ethyl)-1*H*-1,2,3-triazol-4-yl]methyl]acetamide with good radiochemical yield in a one-pot synthesis using nucleophilic ^18^F-fluorination and click chemistry. In vitro studies of curcumin derivative showed specific binding to Aβ, in post-mortem transgenic mouse brain. However, the compound presented low in vivo BBB penetration. Further research is in progress (Table 3).

Yang et al. [107] showed, after in vitro and in vivo studies, that [^18^F]-CRANAD-101 was capable of imaging Aβs in vivo. Therefore, it is a promising candidate for in vivo PET imaging of AD. Specifically, they demonstrated that half-curcuminoid could be a better scaffold for PET tracer development. [^18^F]-CRANAD-101presented significant results to both soluble and insoluble Aβs in the fluorescent spectral tests. The PET measurements showed that 14-month and five-month-old APP/PS1 AD mice (Supplementary Data) presented higher signals in the brain. The half-curcuminoid-based second generation probe [^18^F]-CRANAD-101 exhibited the potential for detecting the early abnormality of the accumulation of Aβs (Table 3).

### 4.2. Imaging Probes for Tau Tangles Detection

Tau aggregation in neuronal cells recently gained significant interest as a robust predictor of the progression of AD. Accordingly, noninvasive imaging of tau aggregates has been highlighted as a promising diagnostic tool for AD.

Boländer et al. [120] synthesizedand evaluated fluorescent pyrazine, pyrimidine, and pyridazine derivatives in vitro and in vivoas possible tau-based diagnostic agents of AD. A pre-evaluation of human brain tissue using fluorescence microscopy was performed. The observationshowed in terms of best contrast and specificity, all the known disease related alterations. 4,4′-(1E,1′E)-2,2′-(pyrimidine-4,6-diyl)bis(ethene-2,1-diyl)bis(*N*,*N*-dimethylaniline) showed a remarkable higher selectivity for aggregated tau, with respect to an early onset diagnosis of AD. Furthermore, the ability of this compound to pass the BBB was demonstrated in a transgenic mouse model by the Aβ binding in vivo (Table 3).

Park et al. [119] developed curcumin-based NIR fluorescent probe of tau fibrils, bystructurally modifying the curcumin scaffold. As prerequisite, the curcumin derivative ought to preserve its binding affinity to the tau fibrils, and as a consequence, the probe should present significant fluorescent properties. To meet these requirements, they developed a new curcumin frame with several substituents on the aromatic rings. Within the series, the curcumin derivative (1E,4Z,6E)-1,7-bis(4-(dimethylamino)-2,6-dimethoxyphenyl)-5-hydroxyhepta-1,4,6-trien-3-one with a (4-dimethylamino-2,6-dimethoxy)phenyl scaffold indicated an important change in its fluorescent properties after binding to tau fibrils. Fluorescence imaging of tau-green fluorescent protein-transfected SHSY-5Y cells (Supplementary Data) with the specific curcumin derivative selectively detected tau fibrils in live cells (Table 3).

**Figure 17:**
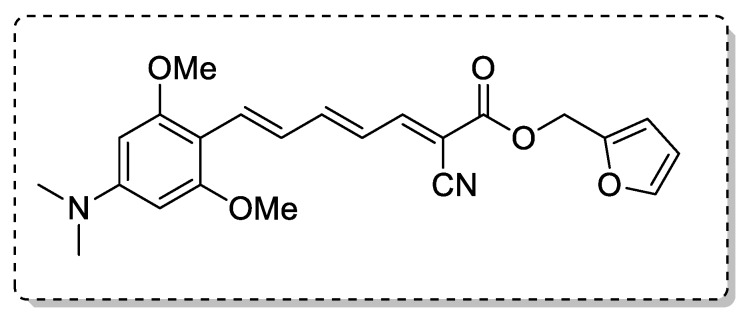
NIRF probe (1) with “turn-on” fluorescence.

A tau–specific “turn-on” NIRF probe (1) (Figure 17) was previously identified, in 2017, byPark et al. [120]. In order to optimize the physicochemical and fluorescence properties, the researchers tried to modify its structure. Thus, they synthesized a number of fluorescent dyes using substituted difluoroboron β–diketonate variously substituted and an *N*,*N*-dimethylaniline moiety linked by a length-extendable π-bridge. The most promising properties as a tau-specific NIRF probe, was shown by6-((1E,3E)-4-(4-(dimethylamino)phenyl)buta-1,3-dien-1-yl)-2,2-difluoro-4-isobutyl-2*H*-1,3,2-dioxaborinin-1-ium-2-uide, which was synthesized byisobutyl-substituted difluoroboron β-ketonate with a π-conjugated 1,4-butadienyl linker. This derivative was compared with NIRF probe (1) andshowed 8.8- and 6.2-times higher tauover Aβ, and tauoverBSA specificity, respectively, and the fluorescence intensity upon binding to tau fibrils was substantially higher (~2.9 times) than (1). The mechanism for tau specificity of the curcumin derivative was investigated. It was suggested that the molecular rotor-like property of the curcumin derivative supports specific recognition of the microenvironment of tau aggregates to emit strong fluorescence. In transgenic cell lines expressing GFP (pCMV6-htau40-green fluorescent protein)tagged tau proteins, the curcumin derivative showed good colocalization with tau-GFP. Moreover, the fluorescence from the curcumin derivative exhibited complete overlap with p-Tau antibody staining in the human AD brain tissue. These results, overall, support the ability of curcumin derivative as a tau-specific fluorescent dye in both in vitro and ex vivo settings (Table 3).

## 5. Summary and Conclusions

**Figure 18:**
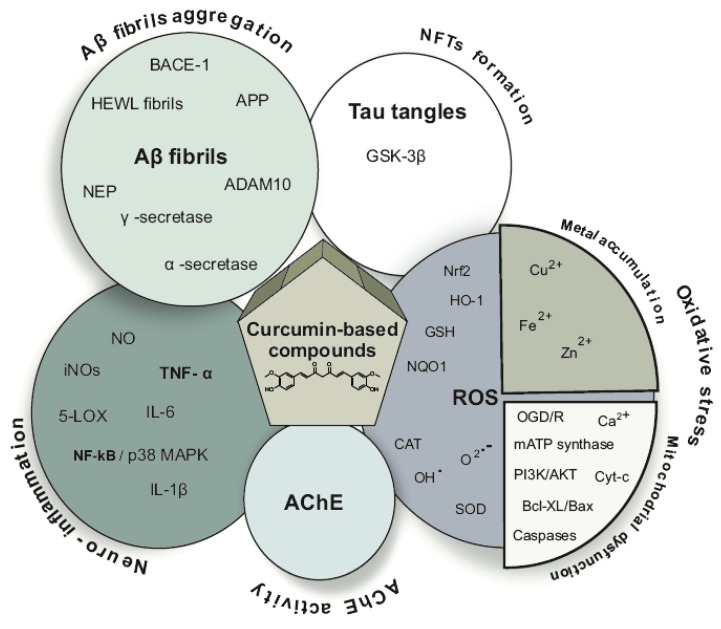
Therapeutic targets of curcumin analogues, derivatives, and hybrids in AD.

AD is the most common type of dementia among the elderly. The percentage of patients is increasing rapidly over the years. To date, no effective cure for the disease has been discovered. However, many efforts have been made by researchers around the world to create a therapeutic compound. Curcumin is a natural pleiotropic substance presenting anti-inflammatory, antioxidative, anti-amyloid activities, as well as inhibition of AChE. Its pleiotropic profile leads to lack of selectivity. The diminished selectivity, in combination with poor bioavailability, are responsible for the limited use. In this context, many innovative curcumin-based compounds have been designed and synthesized over the last decade. Designing and synthesizing new molecules with increased target selectivity, as well as better pharmacokinetic profile, is in progress. In this regard, curcumin derivatives have been designed and synthesized, having anti-amyloidogenic, tau formation inhibitory activity, as well as anti-neuroinflammation, antioxidative, and AChE inhibitory activities (Figure 18). Such molecules are illustrated in Figure 1, Figure 2, Figure 3, Figure 4, Figure 5, Figure 6, Figure 7, Figure 8, Figure 9, Figure 10, Figure 11, Figure 12, Figure 13, Figure 14 and Figure 15. So far, the design of curcumin analogues is more focused on the inhibition of amyloid-β and the suppression of oxidative stress, while fewer efforts have been made to design and synthesize molecules that inhibit both protein accumulation and AChE in the brain.

From the results (Table 1), the presence of phenyl methoxy and hydroxyl groups seems to play a crucial role in inhibiting accumulation of Aβ. Additionally, phenolic groups combined with methoxyl moiety in ortho position and hydroxyl substituent are involved to the oxidative stress suppression (Table 2). It seems that the styryl function and steric or electronic factors through the large aromatic structure are contributed to the antioxidant activity.

Curcumin and its derivatives due to the existence of two aromatic rings and the distance between them, could favorably interact with both the quaternary and peripheral sites of AChE through hydrogen bonds. The modification of the keto-enol moiety is characterized as the best alternative for the design of more potent AChE inhibitors.

Curcumin hybrids, which combine tacrine, donepezil, rivastigmine melatonin with curcumin (AB76, AB77, AB78, AB79, AB80 and AB81) target many factors implicated in Alzheimer’s disease (Figure 16). With the exception ofAB80 and AB81, all the others showed increased permeability to the blood–brain barrier.

Compounds AB3, AB9, AB10, AB14, AB52 present very good results against multiple targets. Cumulative curcumin-based compounds that show activity in more than one target are listed in Table 4.

Diagnostic imaging plays an important role in the diagnosis and treatment of AD. Neurologists and psychiatrists are supported by depended magnetic resonance imaging (MRI) to identify atypical cases, anatomical changes that characterize AD.

Untill now, the best diagnostic technique uses radiotracer [^18^F]FDG. However, it is not effective enough for brain imaging in AD since the diagnosis is made indirectly (glucose levels) and not directly (Aβ plaques or tau tangles) [104]. Curcumin is bound to both soluble and insoluble Aβs. Thus, the curcumin scaffold is a unique structure for second generation Aβ PET tracer development [107]. Many curcumin-based compounds have been tested in vivo in brain imaging with the vast majority being related to the detection and targeting of amyloid-β.

Compounds which are labeled with a radioactive element such as ^68^Ga and ^18^F,[^18^F]-CRANAD-101 especially, have been tested in vivo PET imaging with very promising results and have been able to detect the early abnormality of the accumulation of Aβs (Table 3). The interaction of curcumin-based compounds with Aβ fibrils are also of interest and would be helpful in the design of suitable molecules which simultaneously would provide treatment and diagnosis. It was found that the binding activity of the keto analogue of curcumin to Aβ aggregates was weaker than of theketo-enol tautomers. The keto-enol curcumin tautomers will be the lead for the design of amyloid-binding agents to be used as theranostics in AD. Nanoliposomes decorated with curcumin derivatives, maintained the planar structure and presented increased affinity to the Aβ fibrils. These nanosized curcumin-decorated liposomes showed the highest affinity for Aβ_1–42_ fibrils for in vivo applications.

## Figures and Tables

**Table 1:** Modifications of curcumin’s structure leads to anti-amyloidogenic activity.

Ref.	Modification of Curcumin	Action	In Vitro/In Vivo/Ex Vivo/In Silico	Blood-Brain-BARRIER (BBB) Permeation
**Anti-Amyloidogenic Activity**				
[39]	Optimization of the o-phenol and olefin spacer Incorporates a C5-monoketone spacer moiety and phenolic rings bearing hydroxy groups at positions 2,3,2′,5′	potent anti-amyloid-β aggregation activity	In vitro docking	NI
[18]	Both the 4-hydroxy,3-methoxy and prenyloxy aryl substitution patterns are present	inhibit the formation of large toxic Aβ oligomers	In vitro docking	NI
[7]	Acetylation at only one side of the molecule	strong anti-aggregation effect	In vitro	NI
[40]	Phenolic group combined with methoxyl moiety in ortho position	inhibit Aβ_1–40_ aggregation	In vitro	NI
[19,41]	Replacement of diketone with a pyrazole	potent Aβ aggregation inhibitor	In vitro In vivo	Can pass BBB
[9]	Expansion of the aromatic rings An electron-releasing group to transfer electrons to the phenyl moiety Groups with large conjugated structure or electron-releasing groups	Aβ_1–42_ aggregation inhibition	In vitro	NI
[42,43]	Two aromatic rings connected by a nitrogen containing bridge	block extracellular amyloid toxicity	In vivo (rat hippocampal neurons)	Can pass BBB
[16]	Dimethylaminomethyl-substituted derivatives which have a large steric hindrance, to the *ortho* position of the hydroxy groups	inhibit the Aβ self-aggregation	In vitro	can pass BBB
[44]	Enol form of the compound Methoxycarbonylethyl group at the C-4 position	(i) high affinity for Aβ aggregation (ii) significantly attenuation of the cell toxicity of Aβ	In vitro In vivo	Can pass BBB
[30]	Hydroxyl groups on the aromatic ring Combination of 1,7-bis (4′-hydroxy-3′-trifluoromethoxyphenyl) groups and a suitable substituent at the C-4position	inhibit Aβ aggregation	In vitro	NI
[37]	Phenyl methoxy groups Hydroxyl groups	effect on Aβ_42_, APP and BACE1	In vitro	NI
[45]	Demethoxycurcumin	effect on amyloid-β precursor protein through the internal ribosome entry sites	In vitro	can pass BBB
[35]	Aminoacid (isoleucine, phenylalanine and valine)	α-secretase activation	In vitro	NI
[46]	At least one enone group in the spacer between aryl rings An unsaturated carbon spacer between aryl rings Methoxyl and hydroxyl substitutions in the meta and para-positions on the aryl rings	anti-Aβ aggregation activity	In vitro In vivo	can pass BBB
[47]	Monogalactose group	inhibits Aβpeptide aggregation	In vitro	possibility to optimize the uptake across the BBB via the glucose transporter mechanism
[11]	The introduction of flexible moieties at the linker	inhibit β-sheet aggregation and fibril formation	In vitro	NI
[48]	Modifications in the spacer and the phenolic rings and diketone moiety of curcumin was replaced by cyclohexanone Methoxy substitution of hydroxyl groups Size of these compounds and presence of aromatic/cyclic components	inhibit HEWL aggregation and inhibit the cytotoxic activity of aggregated HEWL	In vitro docking	NI
[49]	The presence of a second –OH group	inhibition of the formation of amyloid-β aggregates	In vitro	NI
[38]	Phenolic hydroxyl group	inhibits the formation of amyloid fibrils	In vitro	NI
[50]	β-diketone moiety, phenolic OH groups, acetyl groups, benzene cycle, hepta-diene moiety and substitutions on the benzene cycle	HEWL aggregation	In vitro docking	NI
[51]	Bisdemethoxycurcumin Diacetylbisdemethoxycurcumin	HEWL aggregation	In vitro docking	NI
[52]	2-methoxy-4-methylphenyl 3,7-diaminoheptanoate	HEWL aggregation	In vitro	NI
[25,53]	Motif Two polar phenolic hydroxy groups and an alkenyl spacer Ketones and double bonds in the spacer O-phenol motif Replacement of phenols with indole and pyrrole	inhibit Aβ aggregation inhibition of BACE-1	In vitro docking	NI
[54]	Contain more hydrophilic hydroxyl groups	upregulating NEP	In vitro In vivo	can pass BBB
[55]	Tetrahydrocurcumin	protective effect against oligomeric amyloid-β induced toxicity	In vitro	NI
[26]	Side aryl rings 4-Hydroxy-3-methoxyphenyl as A ring 4-Benzyloxyphenyl or para-tolyl as B ring	inhibition of BACE-1	In vitro docking	can pass BBB
[56]	Half side of curcumin’s structure	protection against Aβ toxicity through SKN-1/Nrf activation	In vitro	can pass BBB
[57]	Steric, electrostatic and hydrogen bond acceptor, play important role for interaction with the receptor site cavity Interactions between the amino acid residues at the catalytic site of the receptor and the ligands Methylene group (–CH_2_–) in between two carbonyl groups Methylene group attached to the –NH_2_ group The doublebonds of the benzene ring adjacent to the oxygen atom (vi) –CH_3_ of the methoxygroup	Aβ aggregation inhibitory activity	QSAR, pharmacophore modeling, molecular docking and ADME prediction	can pass BBB
[58]	Possessing 2-nitro and 4-dimethylamine groups Having a 4-piperidone Possessing *N*-methyl-4-piperidone	protective against Aβ-induced neuronal cell death	In vitro	NI
[59]	4,4′-((1E,1′E)-(1-phenyl-1*H*-pyrazole-3,5-diyl)bis(ethene-2,1-diyl))bis(2-methoxyphenol)	(i) inhibits neither β- nor γ-secretase activity (ii) induces expression of the ER chaperone glucose-regulated protein 78 (GRP78) (iii) enhances formation of the AβPP/GRP78 complex	In vitro	NI
[60]	Palmitic Acid Curcumin Ester	neuroprotective effects against Aβ insult	In vitro	NI
[29]	Gd(III)(diethylenetriaminepentaacetate) tert-butyl (2-propionamidoethyl)carbamate	redirecting metaltriggered Aβ aggregation	In vitro	can pass BBB
[61]	KLVFFA peptide	strongly inhibit Aβ amyloid fibril formation	In vitro	NI
[62]	Cholesterylamine spacer (length of 17 atoms)	inhibits the formation of amyloid-β oligomers (AβOs)	In vitro	NI
[63]	4-Hydroxyl group	protect from Aβ_1–42_	In vitro	can pass BBB
[33,64]	4,6-Bis((E)-4-(1*H*-imidazol-1-yl)styryl)-2,2-difluoro-2*H*-1,3,2-dioxaborinin-1-ium-2-uide	(i) lowers Aβ levels in conditioned media and reduces oligomeric amyloid levels in the cells (ii) attenuates the maturation of AβPPin the secretory pathway (iii) upregulates α-secretase processing of AβPP and inhibited β-secretase processing of AβPP by decreasing BACE1 protein levels	In vitro In vivo	can pass BBB
[24]	Hydroxyl group	Aβ self-aggregation inhibitory activity	In vitro	can pass BBB
[65,66]	4-OH group	inhibitory effects on the production of amyloid-β oligomers (AβOs)	In vitro In vivo	can pass BBB
[67]	Ortho-methoxy carbamoyl moiety	inhibition of Aβ_1-40_ fibril formation	In vitro	NI
[68]	Presence of electron rich groups on benzyl ring and the terminal benzylpiperidine	inhibition of self-mediated Aβ_1–42_ aggregation	In vitro	possibility to pass BBB
[69]	Feruloyl-donepezil hybrid	ability to modify the kinetics of Aβ fibril formation	In vitro In vivo	can pass BBB

NI, no information is available.

**Table 2:** Oxidative stress properties of curcumin analogues, derivatives, and hybrids in AD.

Ref.	Structure or Functional Groups	Antioxidant Activity	Metal Accumulation	Mitochondrial Dysfunction/Apoptosis	Techniques	In Vitro/In Vivo/Ex Vivo/In Silico	Blood–BrainBarrier (BBB) Permeation
[82]	Mono-ketone moiety Phenolic OH	(i) inhibit ROS accumulation (ii) inhibit oxidative stress via Keap1/Nrf2/HO-1 signaling pathways	-	(i) increase Bcl-2 expression level (ii) decrease the level of Bax and Cyt-c	ROS production assay (DCFH-DA probe)	In vitro (PC12 cells)	NI
[6]	1,7-Bis(3,4-dihydroxyphenyl)-1,6-heptadiene-3,5-dione	reduce intracellular ROS	-	(i) increase the ratio of Bcl-XL/Bax protein level, cytochrome c protein expression (ii) cleave caspase-9 and caspase-3 protein expression	(i) determination of intracellular ROS by DCF assay (ii) Cell apoptosis analysis	In vitro (SK-N-SH cells)	NI
[63]	4-Hydroxyl group	significant decrease in ROS generation and a significant increase in GSH level	-	-	(i) ROS production assay (DCFH-DA probe) (ii) determination of glutathione (GSH)	In vitro (SK-N-SH cells)	pass
[55]	Tetrahydro-curcumin (THC)	increases in the level of reactiveoxygen species	-	(i) decrease in mitochondrial membrane potential, (ii) caspase activation	(i) ROS production assay (DCFH-DA probe) (ii) mitochondrial membrane potential assay (iii) caspase-3 activity assay	In vitro (rat primary hippocampal and human neuron cultures)	NI
[20]	Demethylcurcumin	IncreasesGSH and reduceds reactive oxygen species (ROS)	-	-	(i) ROS production assay (DCFH-DA probe) (ii) glutathione (GSH) assay	In vitro (HT4 neuronal cells)	pass
[60]	Phenol groups	effect on Aβ-induced ROS production in vitro and in vivo	-	-	(i) ROS production assay (DCFH-DA probe) (ii) lipid peroxidation inhibition	In vitro (SH-SY5Y cells) In vivo	NI
[8]	Curcumin congeners with different polyamine motifs	decrease of ROS levels	-	an efficientintracellular uptake and mitochondria targeting	(i) ROS production assay (DCFH-DA probe) (ii) heme oxygenase-1 (HO-1) induction	In vitro (SH-SY5Y, hippocampal neuronal HT22 cell lines, T67 glioma cells)	NI
[42]	(E)-*N*-(2,4-dimethylphenyl)-2,2,2-trifluoro-*N*′-(3-methoxy-benzylidene)acetohydrazide	-	-	-	(ii) heme oxygenase-1 (HO-1) induction	In vivo (APP/PS1 transgenic mice)	pass
[18]	Vanillin moieties 4-Hydroxy,3-methoxy group	(i) reduction of H_2_O_2_-induced intracellular ROS production (ii) activation of antioxidant Nrf2 signaling	-	-	DCFH-fluorescence intensity in H_2_O_2_^-^treated cells	In vitro (SH-SY5Y cells)	NI
[9]	The styryl function and steric or electronic factors through the large aromatic structure A dimethylamino groupon benzene ring	reduction of oxidative stress	selectively chelating metal ions such as copper and iron	-	(i) ORAC (oxygen radical absorbance capacity) (ii) ORAC-FL	In vitro	NI
[11]	The styryl function and steric or electronic factors through the introduction of the piperazine groups	ability to counteract the formation of ROS	chelate metals such as iron and copper	-	(i) ROS production assay (DCFH-DA probe) (ii) ORAC	In vitro (SH-SY5Y cells)	NI
[16]	Phenolic hydroxy groups	-	-	-	DPPH	In vitro	pass
[27,40]	Hydroxyl substituent on the aromatic ring Keto-enolic group A phenolic group combined with methoxyl moiety in ortho position	complexes display possible superoxide dismutase (SOD)-like activity	ability to bind Cu(II) ion	-	(i) DPPH (ii) xanthine/xanthine oxidase assay	In vitro	NI
[87]	Phenolic OH group CH_2_ group of the β-diketone moiety Pyrazole ring andmethoxy groups	enhancing levels of: (i) GSH (mg/g brain tissue) (ii) paraxosnase (kl/mg protein)	-	decrease in brain 8-OHG level, caspase-3 level, and brain p53 level	-	In vitro In vivo (female albino rats)	pass
[22]	4,4′-((1E,1′E)-(1-phenyl-1*H*- pyrazole-3,5-diyl)bis(ethene-2,1-diyl))bis(2-methoxyphenol)	(i) O2^•-^ scavenging activity was much lower than that of curcumin (ii) OH^•^ scavenging activity was similar to that of curcumin	-	-	(i) ORAC (O2^•-^) (ii) HORAC (OH^•^)	In vitro	pass
[39]	Incorporates a C5-monoketone spacer moiety and phenolic rings bearing hydroxy groups at positions 2,3,2′,5′	-	-	-	DPPH	In vitro	
[23]	(1E,4Z,6E)-5-hydroxy-1,7-bis(4-hydroxyphenyl)hepta-1,4,6-trien-3-one	-	-	-	(i) DPPH (ii) FRAP assay	In vitro	NI
[43,88]	(E)-*N*-(2,4-dimethylphenyl)-2,2,2-trifluoro-*N*′-(3-methoxy-benzylidene)acetohydrazide	-	-	exhibits favorable bindingat the allosteric site of *m*ATP synthase with considerable electrostatic energy contributionsfrom Gln215, Gly217, Thr219, Asp312, Asp313, Glu371, and Arg406	-	(i)homology modeling, validation, and active site identification (ii) molecular dynamics (MD) simulation (ii) docking	NI
[89]	7-(4-Hydroxy-3-methoxyphenyl)-1-phenyl-4E-hepten-3-one	-	-	(i)protects cortical neurons fromOGD/R-induced autophagic apoptosis (ii) suppresses OGD/R-induced autophagyand apoptosis in mTOR-dependent manner	OGD/Rinducedcell apoptosis was detected using the Annexin VFITC/PI (propidium iodide) Apoptosis detection kit	In vivo	pass
[81]	A 7-carbon dienone spacer A 5-carbon enone spacer with and without a ring A 3-carbon enone spacer	activation of antioxidant Nrf2 signaling	-	-	DPPH radical scavenger assay	In vitro (Nrf2-ARE reporter-HepG2 stable cell line)	NI
[90]	Bivalent compounds with varied spacer length and cell membrane anchor moiety	-	-	(i) can reverse the increased mitochondrial membrane potential (ii) can abolish the cytosolic Ca^2+^increase upon TC removal	(i) MC65 apoptosis assay (ii) MC65 mitochondrial membrane potential assay (iii) SH-SY5Y mitochondrial membrane potential assay	In vitro (SH-SY5Y andMC65 cells)	NI
[29]	Gd(III) (diethylenetriamine-penta-acetate)-linked-2-(4-((1E,4Z,6*E*)-5-hydroxy-7-(4-hydroxy-3-methoxy-phenyl)-3-oxohepta-1,4,6-trien-1-yl)-2-methoxy-phenoxy)-*N*-(2-(3-(*p*tolyl)thioureido)ethyl)acetamide	-	metal binding properties	-	trolox equivalent antioxidant capacity (TEAC) assay	In vitro (N2a cell line)	NI
[91]	Tetrahydrocurcumin	-	-	tetrahydrocurcumin treatment suppressed neuronal apoptosis in injured brains	-	In vivo	pass
[62]	Cholesterylamine spacer (length of 17 atoms)	antioxidant activity	metal-chelating properties (Cu, Fe, and Zn)	-	ROS production assay (DCFH-DA probe)	In vitro (MC65 cells)	NI
[92]	Bivalent compounds with varied spacer length and cell membrane anchor moiety	-	-	suppress the change of MMP, possibly via interaction with the mitochondrial complex I	(i) MC65 mitochondrial membrane potential assay (ii) SH-SY5Y mitochondrial membrane potential assay	In vitro (MC65 and SH-SY5Y cells)	NI
[26]	(1E,4Z,6E)-1-(4-(benzyloxy)phenyl)-5-hydroxy-7-(4-hydroxy-3-methoxyphenyl)hepta-1,4,6-trien-3-one (1E,4Z,6E)-5-hydroxy-7-(4-hydroxy-3-methoxyphenyl)-1-(p-tolyl)hepta-1,4,6-trien-3-one (1E,4Z,6E)-7-(4-(benzyloxy)phenyl)-5-hydroxy-1-(4-methoxyphenyl)hepta-1,4,6-trien-3-one	ROS NQO1 induction	-	-	(i) ROS production assay (DCFH-DA probe) (ii) assay for NQO1 induction	In vitro (T67 cells)	pass
[65,66]	5-(4-Hydroxyphenyl)-*N*-(2-(5-methoxy-1*H*-indol-3-yl)ethyl)-3-oxopentanamide	possible function against ROS accumulation	-	increased the expression level of complexes I, II, and IV of the mitochondria electron transport chain in the brain tissue of APP/PS1 mice	(i) ROS production assay (DCFH-DA probe) (ii) hydrogen peroxide toxicity	In vitro (MC65 cells, HT22 cells) In vivo	pass
[24]	4-(1-Benzylpiperidin-4-yl)-1-(3-hydroxy-4-methoxyphenyl)butane-1,3-dione	antioxidant activity	metal-chelating properties	-	ORAC-FL	In vitro (PC12 cells)	pass
[68]	Methoxy and hydroxyl groups	antioxidant activity	-	-	ORAC	In vitro	pass
[69,93]	(E)-(1-benzylpiperidin-4-yl)methyl 3-(4-hydroxy-3-methoxyphenyl)acrylate	(i) decreases ROS (ii) increases GSH levels	-	-	(i) ROS production assay (DCFH-DA probe) (ii) glutathione (GSH) assay	In vitro (human neuronal cells) In vivo	pass
[58]	2-Methoxy-4-(piperidin-1-ylmethyl)phenyl dimethylcarbamate	antioxidant activity	metal-chelating properties	-	ABTS assay	In vitro	NI
[94]	Phenolic hydroxyl group	antioxidant activity	metal-chelating properties	-	ORAC	In vitro	NI

NI, no information is available.

**Table 3:** Curcumin-probes as potential therapeutic and diagnostic agents for AD.

Targeting Amyloid-βPlaques				
Ref.	Structure’s Name	Imaging	Effects	Potential Application
[108]	CRANAD-2	NIRF	high affinity for Aβ aggregates	potentially used as a tool for drug screening
[109]	Me-CUR 9	NIRF	detectamyloid-β fibrils with high sensitivity	usefulin vitro amyloid fluorescence sensor
[33]	GRANAD-3	NIRF	capable of detecting both soluble and insoluble Aβ species	potential to have a high impact on AD drug development.
[34]	CRANAD-17	NIRF	capable of inhibiting Aβ_42_ crosslinking induced by copper	potential for AD diagnosis and theraphy
[67]	curcumin-derivative liposomes	NIRF	high affinity for the amyloid deposits, on post-mortembrains samples of AD patients	used asAD theragnostic nanoformulations, to carry therapeutic and/or imaging agents to amyloid deposits in the brain
[110]	BMAOI 14	NIRF	ability to label and detect aggregated amyloid-β (Aβ) peptide as a fluorescent probe	Aβ imaging probes
[109]	CRANAD-28	two-photon imaging	could inhibit the crosslinking of amyloid beta induced either by copper or by natural conditions	could contribute to AD diagnosis and therapy development in the future
[111]	[^125^I] 1,5-diphenyl-1,4-pentadien-3-one derivative	NIRF	high binding affinities with Aβ plaques	potential amyloid imaging agent for the detection of senile plaques in AD
[112]	[^3^H]AB14	Autoradiography	significantly higher specific binding in cortical AD brain tissue	potential radioligands for Aβ plaque neuroimaging
[113]	^68^Ga(CUR)^2+^, ^68^Ga(DAC)^2+^, ^68^Ga(bDHC)^2+^	NIRF	high affinity for amyloid-β plaques	potentially directed to the diagnosis of AD
[114]	^68^Ga(CUR)^2+^ and ^68^Ga(DAC)^2+^	NIRF	affinityto synthetic amyloid-β fibrils	possibility of synthesizing a mixed radioactive/fluorescent pharmacophore that can beexploited as a dual-mode imaging tool.
[4]	[^18^F] 1-(4-fluoroethyl)-7-(4′-methyl)curcumin 1	NIRF	high binding affinity for Aβ_1–42_ aggregates, suitablelipophilicity, specific binding to Aβ plaques in Tg APP/PS-1 mouse brain sections	may be a potential radioligandfor Aβ plaque imaging
[115]	[^18^F] 4′-dimethylamino-4″-(2-(2-fluoroethoxy)ethoxy)curcuminoid	NIR	affinity for senile plaques	for Aβ imaging
[116]	[^125^I] and [^18^F]dibenzylideneacetone derivatives	Autoradiography	affinity towardAβ_1–42_ aggregates	potential new scaffold for amyloid-β imaging probes
[117]	[^19^F] FMeC1	MRI	affinity for senile plaques in human brain sections	detecting Aβ deposition in the brain
[118]	[^18^F]2-[3,5-bis (4-hydroxy-3-methoxystyryl)-1Hpyrazol-1-yl]-*N*-{1-[2-(2-(2-fluoroethoxy)ethoxy)ethyl)-1*H*-1,2,3-triazol-4-yl]methyl}acetamide	PET	high amyloid-β plaque binding	a promising tracer for Aβ imaging
[107]	[^18^F]-CRANAD-101	PE	significant response to both soluble and insoluble Aβs	potentialfor detecting the early abnormality of the accumulation of Aβs
**Targeting Tau-Fibrils**				
[119]	(1E,4Z,6E)-1,7-bis(4-(dimethylamino)-2,6-dimethoxyphenyl)-5-hydroxyhepta-1,4,6-trien-3-one	NIRF	selectively detected tau fibrils	a promising NIRfluorescent probe for noninvasive imaging in patients with AD
[120]	Difluoroboron β–Diketonate Probe	NIRF	specific to tau fibrils	potential as a tau-specific fluorescent dye in bothin vitro and ex vivo settings
[121]	4,4′-(1E,1′E)-2,2′-(pyrimidine-4,6-diyl)bis(ethene-2,1-diyl)bis(*N*,*N*-dimethylaniline)	NIRF	affinity for Tau aggregates	potential for an endoscopic diagnosis of AD in the olfactory system

**Table 4:** Curcumin analogues, derivatives, and hybrids and their anti-Alzheimer activities.

Curcumin-Based Compounds	Aβ Aggregation	Tau Formation	Neuro-Inflammation	Oxidative Stress	AChE	BBB
AB3	+	-	+	+	-	+
AB6	+	+	-	-	-	∆
AB7	+	-	-	+	-	+
AB8	+	-	-	+	-	+
AB9	+	-	+	+	-	+
AB10	+	+	+	+	+	+
AB14	+	-	+	+	+	+
AB15	+	-	-	+	-	-
AB17	+	+	-	+	-	+
AB19	+	-	+	+	-	-
AB21	+	-	+	-	-	-
AB22	+	-	-	+	-	+
AB26	+	-	-	+	-	+
AB29	+	+	-	-	-	+
AB31	+	-	-	+	-	-
AB41	+	-	-	+	+	-
AB42	+	-	-	+	-	-
AB43	+	-	+	+	-	-
AB44	+	+	-	-	-	+
AB46	+	-	-	+	-	-
AB47	+	-	-	+	-	-
AB48	+	-	-	+	-	-
AB52	+	-	+	+	-	+
AB56	-	-	+	+	-	-
AB57	-	-	+	+	-	-
AB69	+	-	-	+	-	-
AB71	-	-	-	+	+	+
AB72	-	-	-	+	+	+
AB73	-	-	-	+	+	+
AB76	+	-	-	+	+	+
AB77	+	-	-	+	+	∆
AB78	+	∆	+	+	-	+
AB79	+	-	+	+	-	+
AB80	+	-	-	+	+	-
AB81	-	-	-	+	+	-

(-), no information is available; (+), activity; and (**∆**), possible activity.

## Supplementary Materials

Supplementary Materials can be found at https://www.mdpi.com/1422-0067/21/6/1975/s1.

## Abbreviations

Aβplaque	β-amyloid plaques or senile plaques
AβPP	Amyloid-β precursor protein
Ach	Acetylcholine
AChE	Acetylcholinesterase
AChEI	Acetylcholinesterase inhibitors
AD	Alzheimer’s Disease
ADAM10	A disintegrin and metalloproteinase domain-containing protein 10
ADAM17	A disintegrin and metalloproteinasedomain-containing protein 17
(ADME)T	Absorption, distribution, metabolism, excretion, and toxicity prediction
AKT	Protein kinase B (PKB)
ApoE	Apolipoprotein E
APP IREs	APP internal ribosome entry site
ATF6	Activating transcription factor 6
ATP	Adenosine triphosphate
ATP5A	Allosteric α subunit of *m*ATP synthase
BACE-1	β-secretase or beta-site amyloid precursor protein cleaving enzyme 1
Bax	Bcl-2-like protein 4
BBB	Blood–brainbarrier
BchE	Butrylcholinesterase
BcL-XL	B-cell lymphoma-extra large
Bcl-2	B-cell lymphoma 2
BDE	O-H proton dissociation enthalpy
BMAC	Boronatedmonocarbonyl analogues of curcumin
BNCT	Boron neutron capture therapy
Brain P53	Tumor protein*p53*
CAAs	Cerebral amyloid angiopathies
Caspases	Cysteine-aspartic proteases
CAT	Catalase
CCC_CV_	Test set using LOO cross validation
CCC_tr_	The concordance correlationcoefficient of the training set
CHOP	C/EBP homologous protein
CNS	Central nervous system
CoMFA	Comparative molecular field analysis
CoMSIA	Comparative molecular similarity indices analysis
Cyt-c	Cytochrome complex
DART TOMFS	Direct analysis in real-time mass spectrometry
DAVID	Database for Annotation, Visualization and Integrated Discovery NIAID, NIH
DFT	Density functional theory
DIPEA	Diisopropylethylamine salt
DNA	Deoxyribonucleic acid
DPPH	2,2-Diphenyl-1-picrylhydrazyl radical scavenging
DTPA	Diethylenetriaminepentaacetic acid
EA	Calculated electronic affinity
elF2a	Eukaryotic translation initiation factor 2 alpha
ER	Endoplasmic reticulum
ERK1/2	Extracellular signal-regulated kinase
[^18^F]FDG	Fluorodeoxyglucose(^18^F)
F	Fisher ratio
F01[C-N]	Frequency of C-N at topological distance of 01, a 2D frequency fingerprints descriptor
FRSA	Free radical scavenging activities
GA-MLR	Genetic algorithme multilinear regression
GFP	pCMV6-htau40-green fluorescent protein
GIAO	Gauge-Independent Atomic Orbital
Glu339	Glutamate 339
Gly230	Glycine 230
GRP78	Glucose-regulated protein 78
GSH	Glutathione
GSK3β	Glycogen synthase kinase 3 beta
HEWL	Hen egg-white lysozyme
HQSAR	Hologram quantitative structure–activity relationship
HO-1	Heme oxygenase 1
i.c.v.	Intracerebroventricular
IL-6	Interleukin 6
IL-1β	Interleukin 1β
iNOS	Inducible NO synthase
IP	Ionization potential
IRE1	Inositol-requiring enzyme 1
Keap1	Kelch-like ECH-associated protein 1
KLVFFA peptide	Aβ-binding motif
LPS	Lipopolysaccharide
mATP synthase	Mitochondrial adenosine triphosphate synthase
MCI	Mild cognitive impairment
MD	Molecular dynamic
MDEC-44	Molecular distance edge between all quaternary carbons
MMPs	Matrix metallopeptidases
MRI	Magnetic resonance imaging
mRNA	Messenger RNA
MTDLs	Multitarget-directed Ligand
mTOR	Mammalian target of rapamycin
NEP	Neprilysin
N_ex_ and N_tr_	The numbers of samples in the training set and external set
NFTs	Neurofibrillary tangles
NIR(F)	Near infrared imaging (fluorescent)
NLs	Nanoliposomes
NMR	Nuclear magnetic resonance
2D NMR	Two-dimensional nuclear magnetic resonance
NO	Nitric oxide
Nrf2	Nuclear factor erythroid 2-related factor 2
NQO1	NAD(P)H:quinone acceptor oxidoreductase 1
OGD/R	Oxygen–glucose deprivation and re-oxygenation
ORAC-FL	Oxygen radical absorbance capacity assay using fluorescein
PEG	Polyethylene glycol
PERK	Protein kinase RNA like endoplasmic reticulum kinase
PET	Positron emission tomography
PI3K	Phosphoinositide 3-kinase
PKC	Protein kinase C
Presenilin	γ-secretase
PSD95	Postsynaptic density protein 95
PS1	Presenilin-1
PS2	Presenilin-2
p38 MAPK	p38 mitogen-activated protein kinase
QSAR	Quantitative structure–activity relationship
Q^2^	Cross-validated R^2^
Q^2^LOO	The square correlation coefficient for leave-one-out cross-validation
RDF090m	Radial distribution function 9.0/weighted by atomic masses, RDF descriptors
RNS	Reactive nitrogen species
ROS	Reactive oxygen species
R^2^	The square of correlation coefficient
R^2^_adj_	The adjusted determination coefficient
R^2^_ex_	Coefficient of determination
R^2^_ncv_	Non cross-validated correlation coefficient
R^2^_pred_	Predictive correlation coefficient
SAMP8	Senescence-accelerated mouse prone 8
sAPPα	Soluble amyloid precursor protein α
SAR	Structure–activity relationship
SEE	Standard errors of estimate
Skn-1	The nematode ortholog of Nrf2
SMD	Steered molecular dynamics
SOD	Superoxide dismutase
SOM	Site of metabolism
TBI	Traumatic brain injury
TC	Tetracycline
TERT	Telomerase reverse transcriptase
ThT assay	Thioflavin T fluorescence assay
TNF-α	Tumor necrosis factor –α
Trp63	Tryptophan 63
Tyr198	Tyrosine 198
WHIM	Weighted holistic invariant molecular
WK.eneg	Non-directional weighted holistic invariant molecular
XBP1	X-box-binding protein-1
5-LOX	5-lipoxygenase
8-OHG	8-hydroxyguanosine
