# Supplementary material for: Curcumin in Health and Diseases: Alzheimer’s Disease and Curcumin Analogues, Derivatives, and Hybrids
Source: Int J Mol Sci. 2020 Mar 13;21(6):1975. doi: 10.3390/ijms21061975 (PMC7139886; doi:10.3390/ijms21061975)
Supplement: Supplementary file 1 [file ijms-21-01975-s001.pdf]

<u>QSARINS:</u> (QSAR-INSUBRIA) is a software for the development and validation of multiple linear regression Quantitative Structure-Activity Relationship (QSAR) models by Ordinary Least Squares method and Genetic Algorithm for variable selection.

<u>Cell lines and mice models:</u>

huAPP/PS1: APP/PS1 mice contain human transgenes for both APP bearing the Swedish mutation and PSEN1 containing an L166P mutation, both under the control of the Thy1 promoter. In these mice, expression of the human APP transgene is approximately 3-fold higher than endogenous murine APP

APPswe/PS1dE9: The APP/PS1 mice express a chimeric mouse/ human amyloid precursor protein (APP) with mutations K594N and M595L linked to Swedish familial AD (Mo/HuAPP695swe) and human PS1 carrying the exon-9edeleted variant associated with familial AD (PS1dE9) Swap HEK293 cells: human *HEK293 cell lines* overexpressing APP

Tg mouse: transgenic mouse (Tg2576). The Tg2576 model is one of the most well characterized, and widely used, mouse models of AD. It overexpresses a mutant form of APP (isoform 695) with the Swedish mutation (KM670/671NL), resulting in elevated levels of Aβ and ultimately amyloid plaques.

rTg4510 mouse model: rTg4510 mice express a repressible form of human tau containing the P301L mutation that has been linked with familial frontotemporal dementia.

SH-SY5Y cells: human neuroblastoma cell line

HMECs: human microvascular endothelial cells

PC12 cells: rat pheochromocytoma cell line

SK-N-SH cells: neuroblastoma cell line

HT4 cells: Mouse hippocampal cell line

MC65 cells: a human neuroblastoma cell line (cellular AD model)

MCF7 cells: a breast cancer cell line

SweAPP N2a cells: Swedish mutant APP overexpressed N2a

T67 cells: human glioma cell line

PBMC: A peripheral blood mononuclear cell is any peripheral blood cell having a round nucleus.
